# Structure and bonding in rhodium coordination compounds: a ^103^Rh solid-state NMR and relativistic DFT study†

**DOI:** 10.1039/d3sc06026h

**Published:** 2023-12-07

**Authors:** Sean T. Holmes, Jasmin Schönzart, Adam B. Philips, James J. Kimball, Sara Termos, Adam R. Altenhof, Yijue Xu, Christopher A. O'Keefe, Jochen Autschbach, Robert W. Schurko

**Affiliations:** a Department of Chemistry & Biochemistry, Florida State University Tallahassee FL 32306 USA rschurko@fsu.edu; b National High Magnetic Field Laboratory Tallahassee FL 32310 USA; c Department of Chemistry, University at Buffalo, State University of New York Buffalo NY 14260-3000 USA jochena@buffalo.edu; d Department of Chemistry & Biochemistry, University of Windsor Windsor ON N9B 3P4 Canada

## Abstract

This study demonstrates the application of ^103^Rh solid-state NMR (SSNMR) spectroscopy to inorganic and organometallic coordination compounds, in combination with relativistic density functional theory (DFT) calculations of ^103^Rh chemical shift tensors and their analysis with natural bond orbital (NBO) and natural localized molecular orbital (NLMO) protocols, to develop correlations between ^103^Rh chemical shift tensors, molecular structure, and Rh–ligand bonding. ^103^Rh is one of the least receptive NMR nuclides, and consequently, there are very few reports in the literature. We introduce robust ^103^Rh SSNMR protocols for stationary samples, which use the broadband adiabatic inversion-cross polarization (BRAIN-CP) pulse sequence and wideband uniform-rate smooth-truncation (WURST) pulses for excitation, refocusing, and polarization transfer, and demonstrate the acquisition of ^103^Rh SSNMR spectra of unprecedented signal-to-noise and uniformity. The ^103^Rh chemical shift tensors determined from these spectra are complemented by NBO/NLMO analyses of contributions of individual orbitals to the ^103^Rh magnetic shielding tensors to understand their relationship to structure and bonding. Finally, we discuss the potential for these experimental and theoretical protocols for investigating a wide range of materials containing the platinum group elements.

## Abstract 2

We present the rapid and robust acquisition of ^103^Rh solid-state NMR spectra for a series of inorganic and organometallic compounds. Relativistic DFT calculations provide relationships between ^103^Rh chemical shift tensors, structure, and bonding.

## Graphical abstract

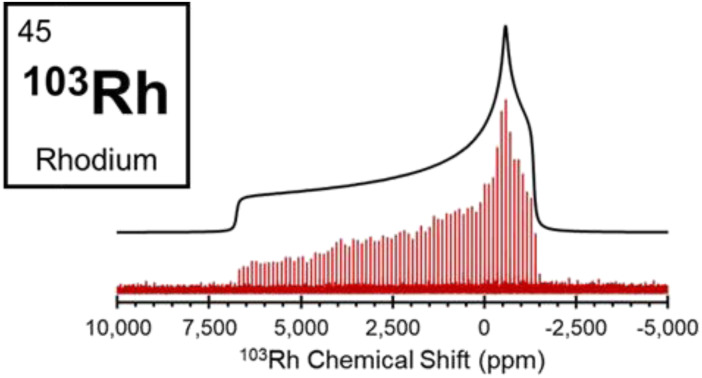

## 1. Introduction

The platinum group elements (PGEs), which include ruthenium, rhodium, palladium, osmium, iridium, and platinum, are scarce and costly, and have serious problems associated with their use, including limited supply chains, recycling, and environmental impact.^1–4^ Despite these problems, PGE-containing materials play fundamental roles in applications such as light generation, solar energy capture, energy materials, nanoparticles, medical implants, coatings, and catalysis, to name only a few.^5^ As such, PGEs have been recognized as *critical materials*.^6^ Rhodium (Rh), the focus of this work, is used extensively in heterogeneous and homogeneous catalysis,^7–9^ including catalytic converters to decrease emissions from automobiles. Rhodium currently stands as the world's most expensive metal, and existing demands for it exceed what can be fulfilled through recycling and new mining sources.^2,3^ Therefore, it is crucial to identify abundant and cheap alternatives to replace PGEs in technologically relevant materials.^10,11^ To do so, questions surrounding what makes PGEs like rhodium special in different contexts must be addressed—this may lead to a rational strategy for replacing PGEs and designing new, advanced materials with tunable properties.

Rhodium coordination compounds are extremely important, with many of their special properties presumably arising from *dative bonding*, *i.e.*, covalent bonding by donation of electrons from coordinating ligands.^12^ It is conceivable that dative bonding in PGE coordination compounds differs in subtle but crucial ways from those of other transition metals, such that it might supersede other influences on structure and reactivity such as ionic radii, electrochemical characteristics, preferred oxidation states, and coordination environments. For instance, the influence of formation and breaking of dative bonds on the electronic ground state potential energy surface must be crucial for a PGE's role in catalytic cycles,^13^ and the combination of covalent and ionic interactions with ligands determine a PGE's function in organometallics, drug products, or vapochromic sensors.^2,13,14^

The combination of solid-state NMR (SSNMR) spectroscopy and first-principles quantum chemical computations using density functional theory (DFT) with a relativistic Hamiltonian provides a pathway for investigating the nature of bonding in rhodium compounds. SSNMR can be applied to investigate ^103^Rh directly, as well as an assortment of nuclides in the bonded ligands.^15–22^ The measurement of the relevant NMR interaction tensors (*e.g.*, ^103^Rh chemical shift tensors) and their calculation provides details about metal–ligand interactions, leading to a deeper understanding of structure–function–property relationships. However, both ^103^Rh SSNMR measurements and DFT calculations of ^103^Rh magnetic shielding tensors present considerable challenges. To date, ^103^Rh chemical shift tensors have been measured for only two materials,^23^ and there have been no computational studies on ^103^Rh magnetic shielding in materials.

^103^Rh is an unreceptive nucleus with a spin of *I* = 1/2, a low gyromagnetic ratio of *γ* = −0.8468 × 10^7^ rad T^−1^ s^−1^, and a natural abundance of 100%.^24^ While there are numerous studies reporting directly- and indirectly-detected ^103^Rh solution NMR spectra,^25,26^ and a small body of literature describing ^103^Rh SSNMR of superconductors,^27,28^^103^Rh SSNMR studies of chemical compounds and materials are very limited. The only directly-detected ^103^Rh SSNMR study of compounds was by Phillips *et al.*, who reported ^1^H–^103^Rh CP/MAS spectra for two Rh complexes, with experimental times ranging from 16 hours to 4 days.^23^ Rossini and co-workers used ^1^H indirect-detection MAS experiments to enhance the sensitivity of SSNMR for low-*γ* nuclei (including ^103^Rh), reporting reductions in experimental times by one to two orders of magnitude in comparison to conventional experiments;^29^ however, these rely on suitable ^1^H–^103^Rh dipolar coupling constants. Experimental challenges arise from the (i) low *γ*(^103^Rh), which leads to low receptivity, small heteronuclear dipolar coupling constants, and the need for probe circuits with reduced acoustic ringing;^30–32^ and (ii) potentially large chemical shift anisotropies (CSAs) that broaden powder patterns. These factors place ^103^Rh among the most challenging nuclides for investigation by SSNMR.

To address the challenges of acquiring static (*i.e.*, stationary sample) SSNMR spectra featuring inhomogeneously broadened, ultra-wideline powder patterns (*i.e.*, hundreds of kHz to several MHz in breadth), our group and others have developed and applied a suite of methods that feature the use of wideband uniform-rate smooth-truncation (WURST) pulses^33^ within the WURST-CPMG^34,35^ and broadband adiabatic inversion cross polarization (BRAIN-CP) pulse sequences (see Section 2.3).^36–39^ These techniques have been applied to an assortment of nuclides with inhomogeneously broadened patterns arising from large CSAs and/or quadrupolar interactions;^37,40–55^ however, they have not been applied to ^103^Rh. In particular, ^1^H–^103^Rh BRAIN-CP experiments have the benefits of providing: (i) theoretical maximum signal gains proportional to *γ*(^103^Rh)/*γ*(^1^H) ≈ 31; (ii) uniform CP efficiency across the entire breadth of the powder pattern (limited only by the excitation and detection bandwidths of the probe); (iii) reduced experimental times due to the dependency of the recycle time on *T*_1_(^1^H) (generally, *T*_1_(^1^H) ≪ *T*_1_(^103^Rh)); and (iv) low rf amplitudes in comparison to conventional CP pulse sequences, which allow for long contact times and reduce wear on solid-state NMR probes.

Relativistic DFT methods increasingly permit calculation of the electronic and magnetic properties of materials containing heavy metal centers, including magnetic shielding tensors that impact NMR spectra.^56^ There are several reports of DFT calculations of ^103^Rh magnetic shielding that highlight the importance of relativistic effects and choice of exchange–correlation functional.^57–66^ However, there are no reports of DFT-based analyses of ^103^Rh magnetic shielding tensors in materials. Recent advances employing the zeroth-order regular approximation (ZORA) relativistic Hamiltonian, combined with cluster-based models, are expected to help address this problem.^67–73^ Furthermore, localized molecular orbital analyses, in their various flavors, provide a rich picture of the relationships between NMR parameters, chemical bonding, and structure. This can be accomplished, for instance, by consideration of contributions of suitably chosen sets of orbitals to the NMR interaction tensors.^74,75^

Herein, we discuss the design and application of ^1^H–^103^Rh BRAIN-CP experiments for the acquisition of ^103^Rh SSNMR spectra using the 21.1 T ultra-wide bore and 36 T series-connected hybrid (SCH) magnets at the National High Magnetic Field Laboratory, and probes adapted for low-*γ* experimentation. The ^103^Rh chemical shift tensors determined from these spectra are complemented by ^103^Rh magnetic shielding tensors obtained from state-of-the-art relativistic DFT-based calculations. Subsequent analyses of contributions of individual localized orbitals (representing bonds, lone pairs, and core shells) to the ^103^Rh magnetic shielding tensors permits exploration of their relationships to structure and bonding. Finally, we discuss the implications of this and future work for better understanding PGE–ligand bonding.

## 2. Experimental and computational details

### 2.1 Samples

All samples were purchased from Strem Chemicals, Inc. and used without further purification. The identities of the bulk materials were confirmed through comparison with simulated PXRD patterns based on known crystal structures (Fig. S1 and S2†).^76–82^ These results indicate that all samples are highly pure; however, in the case of [Rh(NH_3_)_5_Cl]Cl_2_, an additional feature is evident in the ^103^Rh SSNMR spectra (*vide infra*).

### 2.2 Powder X-ray diffraction

PXRD patterns were acquired using a Rigaku Miniflex X-ray diffractometer operating with Bragg–Brentano geometry and featuring a Cu K_α_ radiation source and a D/tex Ultra 250 1D silicon strip detector. The X-ray tube voltage and current were 40 kV and 15 mA, respectively. Samples were packed in zero-background silicon wafers with a well size of 5.0 mm × 0.2 mm, and mounted on an eight-position autosampler. Diffraction patterns were acquired with a detector scanning 2*θ* from 5° to 50° with a step size of 0.03° at a rate of 5° min^−1^.

### 2.3 Solid-state NMR

#### 2.3.1 Overview

SSNMR experiments were conducted at the National High Magnetic Field Laboratory (Tallahassee, FL) using a Bruker Avance NEO console and a home-built 21.1 T ultra-wide bore (105 mm) magnet^83^ and the 36 T Series Connected Hybrid (SCH) magnet (operating at 35.2 T),^84^ corresponding to Larmor frequencies of *ν*_0_(^1^H) = 900 MHz and 1.500 GHz, and *ν*_0_(^103^Rh) = 28.7 MHz and 47.5 MHz, respectively. Home-built 5.0 mm double resonance (HX) low-*E* probes were used for all experiments. The low-*E* design features an outer loop-gap resonator placed around the inner detection solenoid, which permits an increase in the number of turns in the latter, thereby increasing the potential S/N of the low-*γ* detection circuit.^85,86^ All data were collected under static conditions (*i.e.*, stationary samples), with samples packed into cylindrical 5.0 mm o.d. polychlorotrifluoroethylene sample containers designed at MagLab, which reduce ^1^H background signals and allow storage of air-sensitive samples. Sample temperatures were maintained at 25 °C for all measurements. All pulse sequences described herein are summarized in the ESI (Schemes S1–S3†) and are available online at https://github.com/rschurko.

#### 2.3.2 ^1^H–^103^Rh BRAIN-CP experiments: details and practical considerations

The acquisition of ultra-wideline SSNMR powder patterns often involves the use of WURST pulses^33^ for broadband excitation, refocusing, and polarization transfer.^87,88^ In the many cases where the effective transverse relaxation time, *T*^eff^_2_, is favorable, WURST pulses are incorporated into Carr–Purcell Meiboom–Gill (CPMG)-type sequences^89–91^ for the purpose of *T*_2_-based signal enhancement, forming the bases of the WURST-CPMG (WCPMG) pulse sequence for direct excitation,^34,35^ and the broadband adiabatic inversion cross polarization (BRAIN-CP) pulse sequence for indirect excitation.^36–39^ These sequences provide spectra with powder patterns of high intensity and uniformity in comparison to those acquired with conventional rectangular pulses.

All ^103^Rh SSNMR spectra presented herein were obtained using the BRAIN-CP pulse sequence due to its broad excitation, refocusing, and CP bandwidths.^35^ However, the CP-CPMG^92–94^ sequence was used for obtaining signal rapidly over limited bandwidths, which can be useful for locating signals and/or calibrations of rf amplitudes. Direct excitation methods were attempted (*e.g.*, ^103^Rh CPMG and WURST-CPMG) without success; however, CP methods, including CP-CPMG and BRAIN-CP, yielded varying amounts of signal for every sample.

Experimental details related to the BRAIN-CP experiments are provided in Tables S1–S4;† however, parameters most influential to the outcome of the experiments are briefly outlined. The sweep widths of both the contact pulse and excitation/refocusing pulses were set to *ca.* 1.5 to 2 times the target pattern breadth. Due to the slow rate of cross relaxation during CP owing to the low values of *γ*(^103^Rh) and the heteronuclear dipolar coupling, *R*_DD_(^103^Rh, ^1^H),^95,96^ contact times between 10 and 30 ms were used. The maximum amplitude of the ^1^H spin-lock pulse was set to *ν*_2_(^1^H) = 25 kHz for all experiments. A 16-step phase cycling scheme that allows for coherent CP transfer was used for all experiments.^97^ Spectra were acquired with ^1^H continuous wave decoupling with rf amplitudes of 20–25 kHz.

There are several additional factors that can be adjusted in the BRAIN-CP sequence, including the contact time, the shape of the ^1^H spin-lock/contact pulse, and the use of flip-back pulses to reduce recycle delays (Scheme S2†).^39^ Long contact are times are often necessary for low-*γ* nuclides such as ^103^Rh for optimum CP efficiency;^98,99^ however, within the context of a BRAIN-CP experiment, there must be a compromise between the contact time and the number of Meiboom-Gill loops, to reduce the duty cycle of the probe. The application of linearly ramped-amplitude ^1^H spin-lock pulses has the potential to compensate for rf inhomogeneities at offsets far from the transmitter, allowing for the uniform excitation of ultra-wideline patterns (this is especially relevant to the acquisition of ^103^Rh patterns at high fields). Finally, use of a flip-back pulse,^92,100^ which involves the application of an additional π/2 pulse on the ^1^H channel following ^103^Rh acquisition to return the ^1^H transverse magnetization to its equilibrium orientation, has the potential to reduce recycle delays, allowing patterns to be collected more rapidly.

#### 2.3.3 Power calibrations

[Rh(NH_3_)_5_Cl]Cl_2_ was used for the optimization of ^103^Rh rf pulse powers for Hartmann–Hahn matching (*ν*_1,*S*_) and WURST excitation/refocusing (*ν*_exc/ref_) due to its relatively small CSA (*Ω* ≈ 730 ppm) and small *T*_1_(^1^H) time constant (approximated from an optimized recycle delay of 5 s), which allows for short recycle delays. ^103^Rh rf pulse powers were calibrated by arraying the length of a pulse inserted directly after the contact time in the CP-CPMG sequence (Scheme S3†);^92–94^ the pulse width resulting in a null signal was taken as that corresponding to a 90° rotation.

#### 2.3.4 Spectral processing and fitting

^103^Rh chemical shifts were referenced such that *δ*(^103^Rh) = 0 ppm is set using a frequency ratio of *Ξ* = 3.16% relative to the ^1^H resonance frequency of TMS (*l*), as recommended by Carlton.^25,101^ Spectra were processed using a Fourier transformation followed by a magnitude calculation using the Bruker TopSpin v4.1.4 program. Numerical simulations were conducted with the ssNake software package.^102^

### 2.4 Quantum chemical computations

#### 2.4.1 Geometry optimizations

Structural refinements were performed using plane-wave DFT as implemented in the CASTEP module of BIOVIA Materials Studio 2020.^103^ The positions of all atoms were relaxed using the quasi-Newton low-memory BFGS energy-minimization scheme,^104^ in which lattice parameters remained fixed at the given experimental values from X-ray diffraction data (Table S5†).^76–82^ Calculations used the PBE functional,^105^ a plane wave cutoff energy of 800 eV, a *k*-point spacing of 0.05 Å^−1^,^106^ the ZORA Hamiltonian with ultrasoft pseudopotentials generated on the fly,^107^ and an SCF convergence threshold of 5 × 10^−7^ eV. Dispersion was included through the many-body dispersion force field of Tkatchenko *et al.*^108^ Thresholds for structural convergence include a maximum change in energy of 5 × 10^−6^ eV per atom, a maximum displacement of 5 × 10^−4^ Å per atom, and a maximum Cartesian force of 10^−2^ eV Å^−1^.

#### 2.4.2 Magnetic shielding calculations

^103^Rh magnetic shielding tensors were calculated on energy-minimized structural models obtained from geometry optimization. Magnetic shielding tensors were calculated using the gauge-invariant projector-augmented wave (GIPAW) approach in CASTEP,^109^ with the same parameters and approximations used in the aforementioned geometry optimizations. The convergence of these calculations with respect to plane-wave cutoff energy and *k*-point spacing is illustrated in Table S6.† Additionally, magnetic shielding tensors were calculated using the gauge-including atomic orbital (GIAO) method,^33,34^ as implemented in the Amsterdam Modeling Suite (AMS 2021.106 or 2022.102).^110–112^ For calculations employing AMS, an isolated molecule or a cluster of molecules (based on the energy-minimized solid-state structure) was used as a structural model. The GGA functional PBE^105^ and hybrid functional PBE0^113^ were both used, along with the ZORA relativistic Hamiltonian either at the scalar (SR) or scalar + spin–orbit (SO) levels.^114–118^ PBE0, in particular, has been benchmarked extensively in NMR applications and is known to deliver accurate results.^119^ A correction to the exchange–correlation response kernel was implemented in PBE/PBE0 calculations employing the ZORA/SO Hamiltonian.^120^ The Slater-type basis sets TZ2P, DZ, and SZ were used for different regions of the clusters as described below.^121^ Becke integration quality was set to “good” for the TZ2P regions of the clusters and to “normal” for the DZ and SZ regions.^122,123^

A cluster of molecules was constructed to represent the local environment of the crystal lattice for each system, as described in previous work on organic and inorganic molecular solids (Fig. S3†).^73,124–126^ Clusters consist of a central molecule for which the ^103^Rh magnetic shielding tensors is computed, as well as eight to fourteen peripheral molecules that constitute the complete first coordination shell. These calculations used a three-layer basis set partitioning scheme in which the TZ2P basis set was used for the central rhodium and all directly bound atoms, the DZ basis set was used for all other atoms within the same molecular unit, and the SZ basis set was used for all of the atoms in the peripheral molecules. Calculations on isolated molecules using locally-dense (*i.e.*, using TZ2P and DZ for different regions of the molecule) and balanced (*i.e.*, using TZ2P for the entire molecule) basis sets are provided in Table S7.†

#### 2.4.3 NLMO analysis

^103^Rh magnetic shielding tensors were analyzed in terms of contributions from NBOs and NLMOs,^127^ according to previously published protocols.^75,128,129^ These calculations were performed in AMS at the hybrid DFT (PBE0) and SO level for all isolated complexes and clusters of molecules, with other parameters identical to those described above. In each case, an initial set of canonical orbitals was calculated at the ZORA/SR level, followed by localization *via* the NBO 6.0 program included in the AMS package. This was followed by a self-consistent DFT calculation at the SO level. The shielding tensor at the ^103^Rh nucleus was then calculated using the NMR module as implemented in AMS and analyzed in terms of contributions from the full set (occupied and unoccupied) of NBOs and NLMOs. NLMO visualizations are in the form of isosurfaces at ±0.03 atomic units.

## 3. Results and discussion

### 3.1. ^103^Rh SSNMR

#### 3.1.1 Overview

**Scheme 1:**
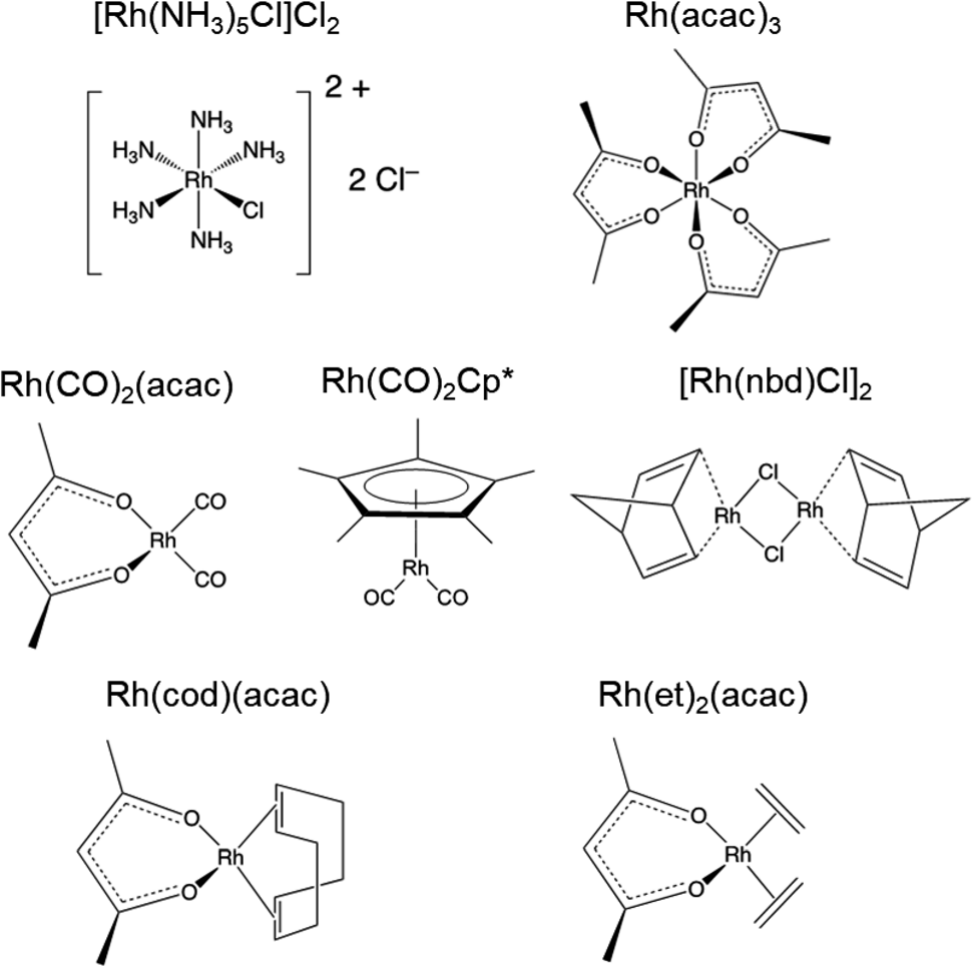
Molecular structures of rhodium coordination compounds.

Seven compounds were chosen for investigation based on the following criteria: (i) they are diamagnetic Rh(i) and Rh(iii) complexes; (ii) they have a Rh wt% above *ca.* 25%, to maximize the number of ^103^Rh spins; and (iii) they have hydrogen atoms, which allows for ^1^H–^103^Rh BRAIN-CP and CP-CPMG experiments. In addition, the compounds were selected to represent a range of structural motifs common to rhodium coordination chemistry, including ligands such as acetylacetonate, amine, chlorine (bridging and terminal), and an assortment of π-coordinating ligands. These compounds include two Rh(iii) complexes, [Rh(NH_3_)_5_Cl]Cl_2_ and Rh(acac)_3_, and five Rh(i) complexes, Rh(CO)_2_(acac), Rh(CO)_2_Cp*, [Rh(nbd)Cl]_2_, Rh(cod)(acac), and Rh(et)_2_(acac) (acac = acetylacetonate; Cp* = C_5_Me_5_^−^; nbd = norbornadiene; cod = cyclooctadiene; et = ethene; all are pictured in Scheme 1).

**Fig. 1:**
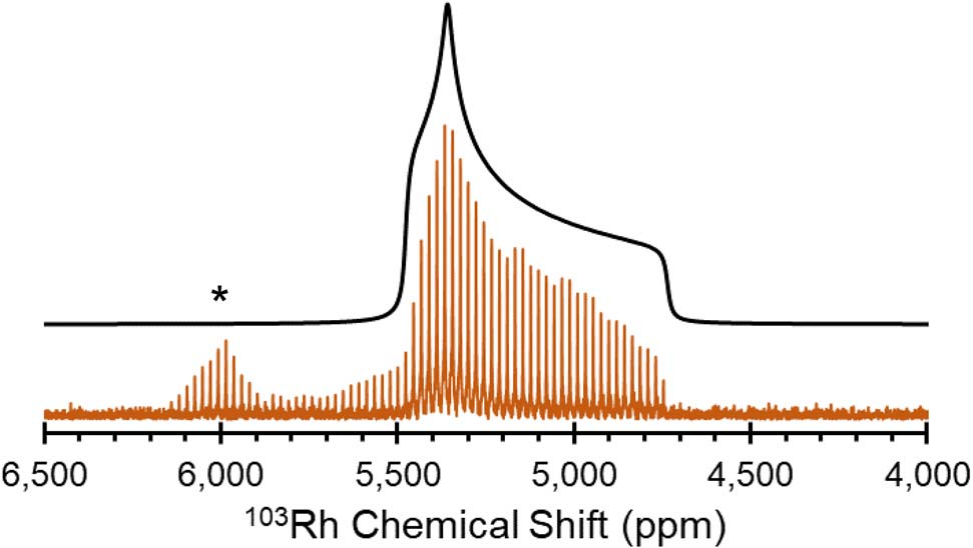
^1^H–^103^Rh BRAIN-CP spectrum of [Rh(NH_3_)_5_Cl]Cl_2_ acquired at 21.1 T and corresponding simulation. An impurity phase is indicated by an asterisk.

[Rh(NH_3_)_5_Cl]Cl_2_ serves as a standard for calibration of ^103^Rh rf amplitudes (*vide supra*), due its short value of *T*_1_(^1^H) (estimated to be <1 s), which enables a relatively short recycle delay of 5 s, and the narrow breadth of its ^103^Rh powder pattern (*ca.* 20 kHz at 21.1 T). These factors allow for the acquisition of ^1^H–^103^Rh CP-CPMG or BRAIN-CP spectra within minutes (Fig. 1). An unexpected pattern is observed to high frequency of the powder pattern corresponding to [Rh(NH_3_)_5_Cl]Cl_2_, the origin of which is unknown. The additional pattern was present in the spectra of multiple “as-received” batches of [Rh(NH_3_)_5_Cl]Cl_2_ as well as samples obtained from recrystallization, and no impurity phases are readily apparent in the PXRD pattern (Fig. S1†). This pattern may correspond to any of a myriad examples of octahedral Rh(iii) chloroamines of the form [RhCl_*x*_(NH_3_)_6−*x*_]·*x*H_2_O, or even to a synthetic precursor such as RhCl_3_·*x*H_2_O.^130,131^ Despite this, the chemical shift tensor parameters can be determined for [Rh(NH_3_)_5_Cl]Cl_2_ (Table 1), and these samples are adequate for purposes of calibration.

**Table 1:** Experimental and calculated ^103^Rh chemical shift tensors, as well as the chemical shift distances, and corresponding solution-state chemical shifts^a^^,^^b^^,^^c^^,^^d^

Material		*δ*_iso_ (ppm)	*Ω* (ppm)	*κ*	*d*_v_ e (ppm)	*δ* (soln.) f (ppm)
[Rh(NH_3_)_5_Cl]Cl_2_	Exp.	5110(30)	730(50)	0.68(0.06)	—	n/a
	Calc.	5012	848	0.81	106	—
Rh(acac)_3_	Exp.	8380(30)	500(30)	−0.68(0.06)	—	8358
	Calc.	8399	554	−0.76	26	—
Rh(CO)_2_(acac)	Exp.	550(80)	2950(100)	−0.20(0.03)	—	292
	Calc.	755	2598	0.07	253	—
Rh(CO)_2_Cp*	Exp.	−1150(80)	1850(100)	−0.05(0.08)	—	n/a
	Calc.	−1150	1874	−0.01	13	—
[Rh(nbd)Cl]_2_	Exp.	1580(50)	7950(150)	−0.81(0.03)	—	n/a
	Calc.	1346	7875	−0.79	238	—
Rh(et)_2_(acac)	Exp.	1150(50)	7100(120)	−0.59(0.03)	—	1167–1262
	Calc.	1164	7448	−0.67	148	—
Rh(cod)(acac)	Exp.	1280(50)	7030(120)	−0.67(0.03)	—	1287–1294
	Calc.	1374	7249	−0.64	110	—

a The chemical shift tensors are defined with the principal components ordered from highest to lowest frequency as *δ*_11_ ≥ *δ*_22_ ≥ *δ*_33_. The Herzfeld–Berger convention is also used herein, where the isotropic chemical shift, span, and skew are given by *δ*_iso_ = (*δ*_11_ + *δ*_22_ + *δ*_33_)/3, *Ω* = *δ*_11_ − *δ*_33_, and *κ* = 3(*δ*_22_ − *δ*_iso_)/*Ω*, respectively.

b The experimental uncertainties for each value are indicated in parentheses.

c Calculations were performed using the hybrid PBE0 functional, the ZORA spin–orbit relativistic treatment, and a cluster of molecules representing the local lattice environment. See the main text for further details.

d The following abbreviations are used in compound names: acac = acetylacetonate; Cp* = C_5_Me_5_^−^; nbd = norbornadiene; cod = cyclooctadiene; et = ethene.

e Chemical shift distance between the experimental and calculated chemical shift tensors. See ESI S1 for definitions.

f Solution-state chemical shifts are reported in a review by Carlton (see ref. 25).

#### 3.1.2 ^103^Rh SSNMR spectra and properties of the chemical shift tensors

**Fig. 2:**
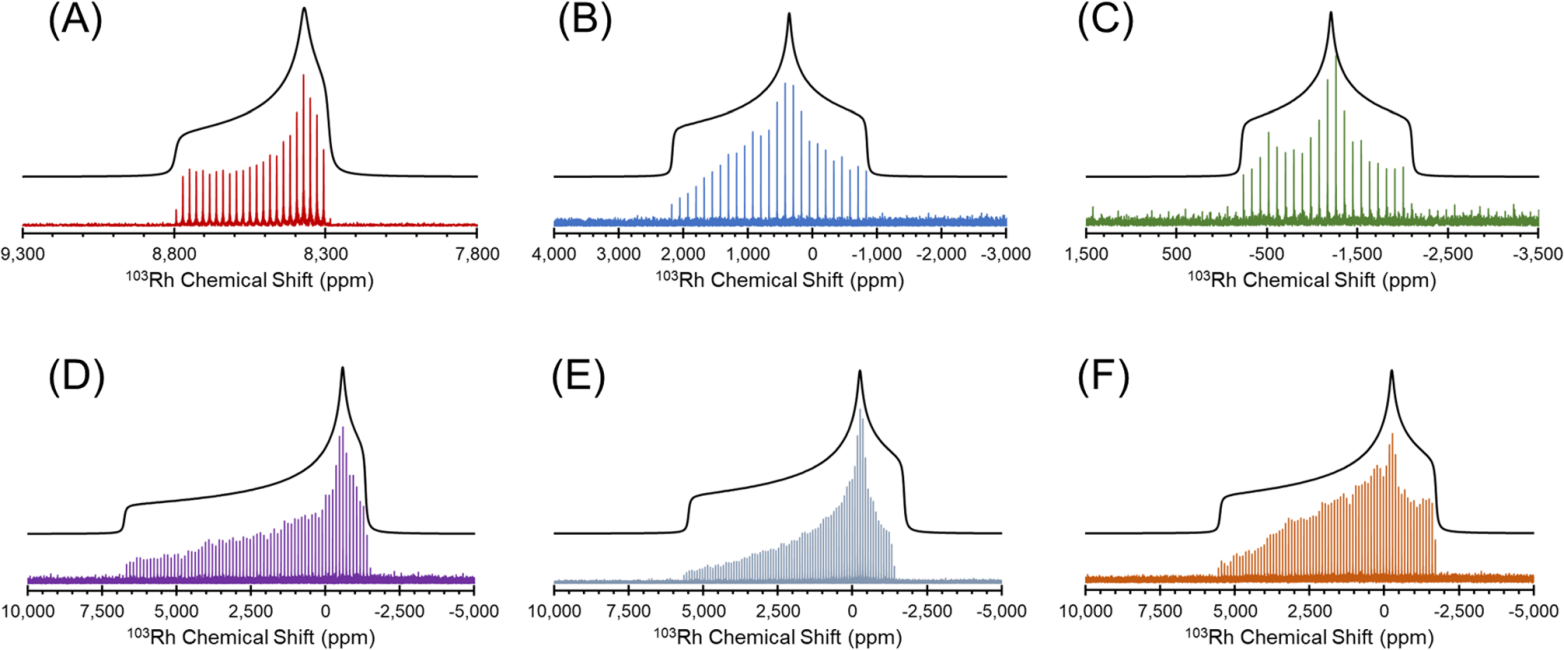
^1^H–^103^Rh BRAIN-CP spectra acquired at 21.1 T for (A) Rh(acac)_3_; (B) Rh(CO)_2_(acac); (C) Rh(CO)_2_Cp*; (D) [Rh(nbd)Cl]_2_; (E) Rh(cod)(acac) and (F) Rh(et)_2_(acac).

The ^1^H–^103^Rh BRAIN-CP NMR spectra of six Rh coordination compounds acquired at 21.1 T, along with best fit simulations, are shown in Fig. 2, with their respective chemical shift tensor parameters in Table 1. Pattern breadths range between *ca.* 15 kHz and 230 kHz, and there are no obvious signs of impurity phases. The spectra consist of a series of “spikelets” that result from the Fourier transform of the CPMG echo train in the time domain. The spectra of Rh(CO)_2_(acac) and Rh(CO)_2_Cp* have lower S/N ratios than the other patterns, because of poor ^1^H–^103^Rh CP efficiency (due to CO ligands that do not contain protons) and/or low values of *T*_2_(^103^Rh).

The ^103^Rh chemical shift tensors, determined from simple analytical simulations of powder patterns that match the outer manifold of the spikelet patterns, reflect the oxidation state and coordination environment of the rhodium atoms, and offer insight into the nature of metal–ligand bonding. Hence, the ensuing discussion features small groups of Rh complexes with similar structural properties.

[Rh(NH_3_)_5_Cl]Cl_2_ and Rh(acac)_3_, which feature six-coordinate Rh(iii) environments, have values of *δ*_iso_ = 5110 ppm and 8380 ppm_,_ respectively; to within experimental error, the chemical shift of solid Rh(acac)_3_ is identical to that of the compound dissolved in CHCl_3_.^25^ The Rh(iii) compounds also have relatively narrow, CSA-dominated powder patterns, with spans of *Ω* = 730 and 500 ppm, and skews of *κ* = +0.68 and −0.68, respectively. The values of *κ* indicate chemical shift tensors of near-axial symmetry, with *δ*_33_ and *δ*_11_ as the distinct components, respectively. For Rh(acac)_3_, the unique principal component is oriented near pseudo-*C*_3_ rotational axis, whereas for [Rh(NH_3_)_5_Cl]Cl_2_, the orientation of the unique principal value is not constrained as such, possibly due to variation in the Rh–N bond lengths (the orientations of these tensors derived from DFT calculations are shown in Fig. S4†).

The remainder of the complexes feature Rh(i) in a variety of coordination environments. *δ*_iso_ values are observed to occur over a range from *ca.* −1150 to 1580 ppm, indicating that these ^103^Rh nuclei are much more shielded than those in the Rh(iii) compounds, again consistent with solution NMR measurements.^25^ However, the Rh(i) complexes have *Ω* values that are considerably larger than those of the Rh(iii) complexes. For the four-coordinate Rh(i) site in Rh(CO)_2_(acac), *Ω* is 2950 ppm, with its moderate *κ* = −0.20 indicative of three distinct principal components. In Rh(i) complexes with η^2^-coordinated alkenic ligands, values of *Ω* range from *ca.* 7000 to 8000 ppm, and negative *κ* values that indicate that *δ*_11_ is the distinct component in each case. For Rh(et)_2_(acac) and Rh(cod)(acac), which both feature a bidentate acac ligand and two η^2^-coordinating ligands, the values of *Ω* and *κ* are similar; however, for [Rh(nbd)Cl]_2_, a higher *Ω* is observed, perhaps due to the presence of bridging Cl ligands. Rh(CO)_2_Cp*, the only compound herein with an η^5^-coordinated cyclopentadienyl ring, has a *δ*_iso_ = −1150 ppm, the lowest in this set, which is indicative of an extremely shielded ^103^Rh nucleus as is typical for *δ*_iso_ values of metal nuclides in metallocenes.^132–135^ The relationships between the ^103^Rh chemical shift tensor parameters and structure and bonding in this set of Rh complexes is further explored with DFT calculations (see Sections 3.2 and 3.3).

#### 3.1.3 Design of ^1^H–^103^Rh BRAIN-CP experiments

**Fig. 3:**
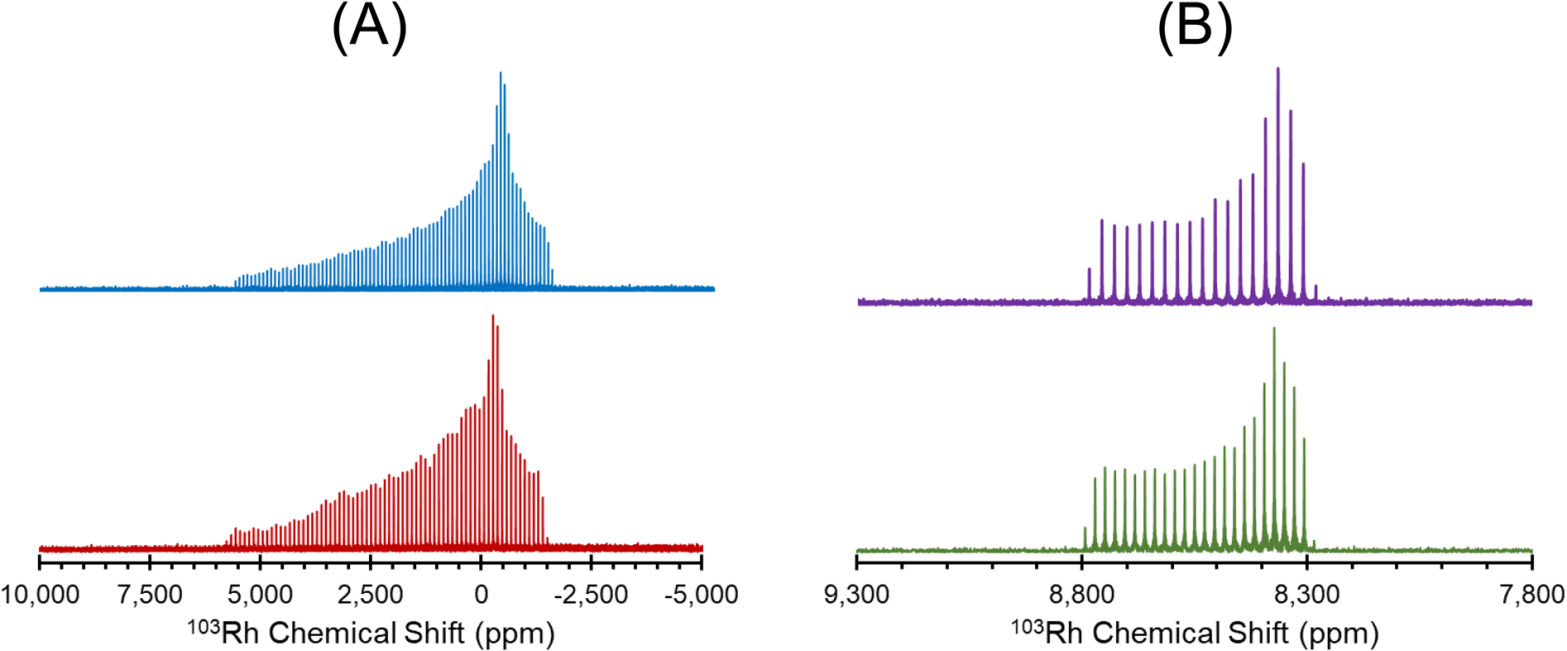
(A) ^1^H–^103^Rh BRAIN-CP spectra of Rh(cod)(acac) acquired at 21.1 T, using (blue) a 30 ms contact time, a ramped amplitude ^1^H spin-lock pulse, and a flip-back pulse to reduce the recycle delay (total experiment time: 6 h), and (red) a 16 ms contact time, a constant-amplitude ^1^H spin-lock pulse, and no flip-back pulse (total experiment time: 17 h). (B) ^1^H–^103^Rh BRAIN-CP spectra of Rh(acac)_3_ acquired at 35.2 T (purple) and 21.1 T (green). Both spectra in B were acquired with the same number of scans, relaxation delay, contact time, Hartman–Hahn matching conditions, and feature similar spikelet spacings.

Carefully calibrated ^1^H–^103^Rh BRAIN-CP pulse sequences permit the acquisition of high-quality static ^103^Rh SSNMR spectra at 21.1 T in reasonable timeframes. Following these initial experiments, we examined additional methods for obtaining ^103^Rh powder patterns with greater rapidity and efficiency, including modifications to the BRAIN-CP pulse sequence and use of higher fields. Experiments implementing long contact times, linearly-ramped ^1^H spin-lock pulses, and ^1^H flip-back pulses are promising (Fig. S5†). For example, the pattern of Rh(cod)(acac) acquired with a 30 ms contact time, a ramped-amplitude spin-lock pulse, and a flip-back pulse is more uniformly excited, features higher S/N, and was acquired in a significantly shorter time frame (6 h *vs.* 17 h) than a similar pattern acquired with a 16 ms contact time, a constant-amplitude spin-lock pulse, and no flip-back pulse (Fig. 3A). Additionally, ^1^H–^103^Rh BRAIN-CP spectra were acquired at 35.2 T for three samples (Fig. S6†). The spectrum for Rh(acac)_3_ suggests that high-quality ^103^Rh powder patterns at 35.2 T can be obtained in similar timeframes as those obtained at 21.1 T (Fig. 3B). However, magnet time at 35.2 T is limited and costly; despite this, and the fact that the breadths of ^103^Rh patterns increase proportional to *B*_0_ (thereby reducing inherent S/N ratios), the gain in S/N (∝*B*_0_^3/2^) and the increased pattern breadths may be valuable for obtaining refined chemical shift tensor parameters. In the present case, increasing *B*_0_ from 21.1 T to 35.2 T is anticipated to increase S/N by *ca.* 29%. These results are promising, and may afford future opportunities for rapid acquisition of undistorted ultra-wideline ^103^Rh SSNMR spectra at fields as high as 35.2 T.

### 3.2 Quantum chemical calculations of ^103^Rh magnetic shielding tensors

For materials comprising heavier elements such as rhodium, the other PGEs, and many of the other transition metal and main group elements, there are significant challenges associated with accurate calculations of magnetic shielding tensors. For example, relativistic effects must usually be treated at the spin–orbit level in DFT calculations to obtain good agreement with experimental chemical shift tensors.^56^ There are also cases in which computed magnetic shielding tensors depend critically on the choice of exchange–correlation functional; in particular, the difference in performance of non-hybrid and hybrid functionals has been noted for several elements, and use of the latter has been noted to be important for rhodium.^58^ Additionally, when calculating NMR interaction tensors in solids, long-range intermolecular effects must often be included to obtain agreement with experiments for the right reasons. These factors necessitate significant computational resources to make such calculations tractable.

Intermolecular effects on ^103^Rh magnetic shielding tensors are modeled accurately in calculations through use of periodic boundary conditions (*e.g.*, using the GIPAW approach). These effects can also be modeled in non-periodic calculations (*e.g.*, with GIAO basis sets) through the use of suitable non-empirically designed clusters of molecules to represent the local lattice environment. Clusters are constructed to represent a complete coordination shell of molecules around a central molecule (*N.B.*: the ^103^Rh magnetic shielding tensor is calculated only for the central molecule) following procedures established in previous studies of molecular solids.^73,124–126^ Two important advantages that cluster-based approaches maintain over popular periodic plane-wave methods include the ability to implement (i) more advanced computational methods such as hybrid DFT with a reasonable computational cost, and (ii) more rigorous relativistic treatments that include spin–orbit coupling. These considerations are all important for obtaining agreement with experimental chemical shift tensors for the right reasons, as well as for predicting electronic structures from which further analyses can produce meaningful relationships between bonding and chemical shifts. It is important to stress here that calculations at the best theoretical level available must be proven to deliver accurate NMR interaction tensors in comparison to state-of-the-art measurements such that the theory-derived electronic structure–property relationships are meaningful and reliable, and will stand the test of time.

The importance of intermolecular effects on ^103^Rh magnetic shielding tensors can be assessed through comparison of calculations using a cluster *vs.* those using an isolated molecule (Fig. S7†). At the PBE0/SO level (hybrid functional), we find that the intermolecular contributions to individual principal components of the magnetic shielding tensor are less than *ca.* 100 ppm for the majority of rhodium species. However, for the Rh(iii) coordination complex [Rh(NH_3_)_5_Cl]Cl_2_ and the Rh(i) planar coordination compound Rh(CO)_2_(acac) that features relatively small Rh–Rh contacts (*r*_Rh–Rh_ = 3.15 Å),^77,136^ intermolecular contributions are as large as *ca.* 1000 ppm for individual principal components; this demonstrates that in some cases, lattice effects are essential to achieve agreement between theory and experiment.

**Fig. 4:**
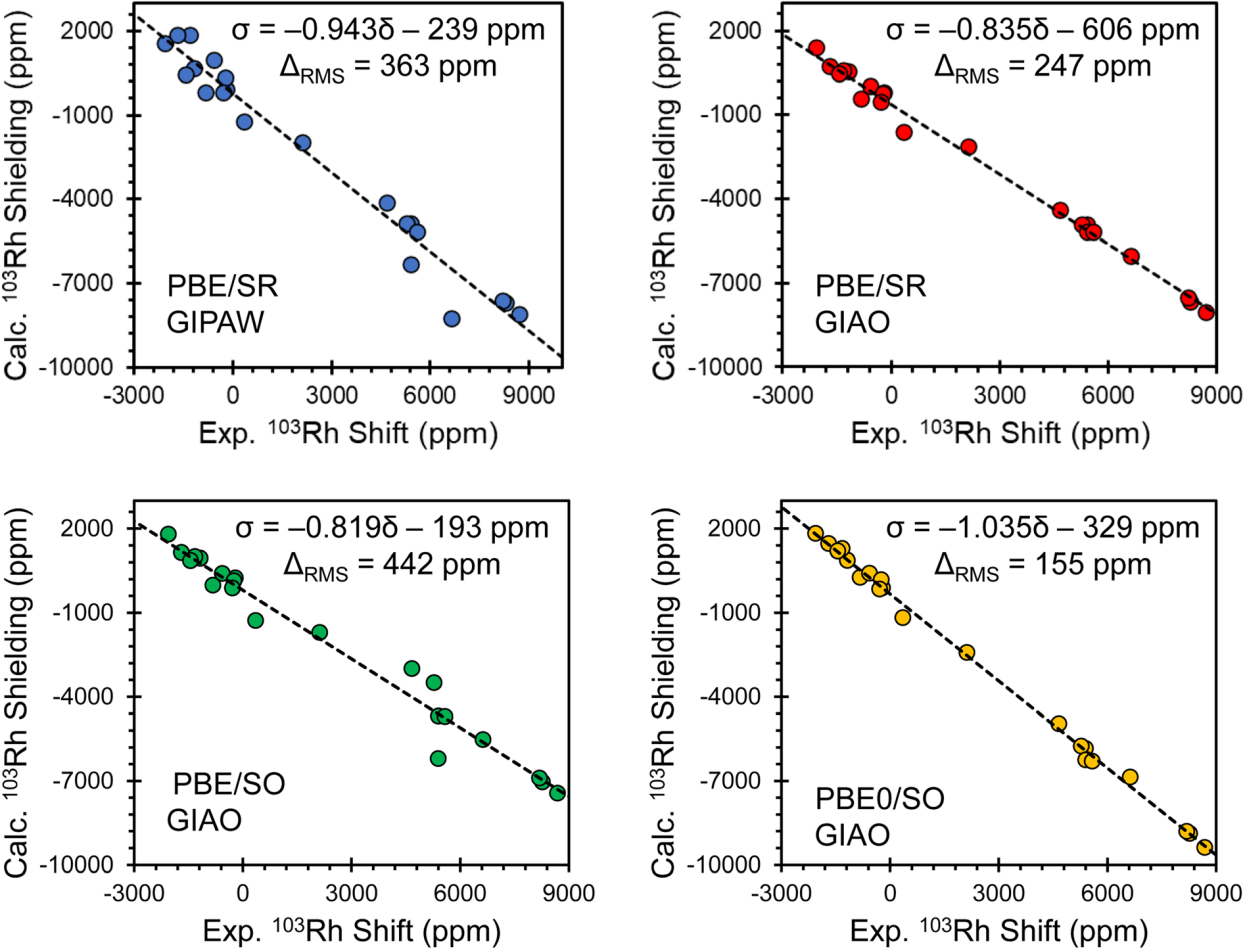
Correlations between calculated principal components of ^103^Rh magnetic shielding tensors and experimental ^103^Rh chemical shift tensors, as determined with different computational protocols. Computed shielding constants were obtained using the theoretical methods as indicated. Black lines represent the best fits, with the equations provided.

Comparisons of principal components of the ^103^Rh chemical shift tensors obtained from experiment and calculated magnetic shielding tensors obtained using several computational methods are shown in Fig. 4, with corresponding lists of calculated parameters in Table S8.† Each panel shows the correlation between experimental values and those obtained from one of four different computational protocols, including one with PBE/SR GIPAW calculations (periodic boundary conditions, scalar-relativistic) and three with GIAO calculations on clusters (using different exchange–correlation functionals and relativistic approximations: PBE/SR, PBE/SO, and PBE0/SO). The relationships between the principal components of experimental ^103^Rh chemical shift tensors and principal components of calculated ^103^Rh magnetic shielding tensors are modeled through least-squares linear regression, with errors provided by the RMS chemical shift distance, *Δ*_RMS_ (see ESI S1 for details†).^137^ The best theory–experiment correlation (slope of −1.035; *Δ*_RMS_ of 155 ppm; yellow plot in Fig. 4 and calculated values in Table 1) is achieved with the following criteria: (i) clusters of up to fifteen molecules are used to account for intermolecular interactions; (ii) the ZORA Hamiltonian, expanded to include SO terms, is used; and (iii) the hybrid PBE0 functional is used. In contrast, the other types of calculations yield slopes between −0.819 and −0.943 (*i.e.*, significant deviations from the ideal value of −1.000) and *Δ*_RMS_ between 247 ppm and 442 ppm. These findings are largely consistent with recent reports of calculations of the chemical shift tensors of other fifth- and sixth-period elements using comparable computational methods.^67–72^

### 3.3 NLMO/NBO analysis of ^103^Rh magnetic shielding tensors

Table 2 summarizes the calculated NLMO contributions to the ^103^Rh isotropic shielding for the complexes isolated from their crystal environment (*i.e.*, isolated molecules). The large differences in total shielding among the complexes are primarily determined by the paramagnetic shielding contributions from the Rh 4d shell, as well as ligand orbitals that directly overlap with the metal valence shells. The Rh core contributions, while not identical, are similar for all complexes, as expected. Also included in the analysis are Rh–ligand bonding interactions (Rh–X). Summarized under “Other” are additional contributions arising from all other occupied orbitals, as well as unoccupied orbital contributions. The full set of NLMO isosurfaces and corresponding labels used throughout the text are found in Fig. S8–S16.† A complete analysis of NLMO contributions are found in Tables S9–S11.†

**Table 2:** Summary of NLMO contributions to ^103^Rh isotropic shielding for isolated rhodium complexes (absent crystal embedding)

	[Rh(NH_3_)_5_Cl]Cl_2_ (1)	Rh(acac)_3_ (2)	Rh(CO)_2_(acac) (3)	Rh(CO)_2_Cp* (4)	[Rh(nbd)Cl]_2_ (5)	Rh(et)_2_(acac) (6)	Rh(cod)(acac) (7)
Rh 4d	−2857 [*1a*: d_*yz*_]	−4245 [*2a*]	−943 [*3a*]	−1007 [*4a*]	−1001 [*5a*]	−862 [*6a*: d_*z*^2^_]	−852 [*7a*: d_*z*^2^_]
	−2846 [*1b*: d_*xz*_]	−4253 [*2b*]	−791 [*3b*]	−581 [*4b*]	−3451 [*5b*]	−3495 [*6b*: d_*xy*_]	−3339 [*7b*: d_*xy*_]
	−2970 [*1c*: d_*xy*_]	−3986 [*2c*]	−2134 [*3c* + *3d*]^d^	−947 [*4c*]	−523 [*5c*]	−580 [*6c*]	
				−463 [*4d*]		−366 [*6d*]	
∑ above 4d^b^	−8673	−12483	−3868	−2998	−4975	−5303	−4191
Rh core	4238	4384	4226	4172	4355	4383	4352
Rh–X	−241 [*1d*(5)]^c^	−510 [*2d*(6)]^c^	−464 [*3e*(2)]^c^	36 [*4e*(2)]^c^	−436 [*5d*(2) + *5e*]^c^	−338 [*6e*(2)]^c^	−300 [*7c*(2)]^c^
	−25 [*1e*]	−60 [*2e*(3)]^c^	−316 [*3f*(2)]^c^	−422 [*4f*(2)]^c^	−459 [*5f*(4)]^c^	−140 [*6f*(2)]^c^	−1529 [*7d*(2) + *7e*(2)]^c^
						−18 [*6g*]	−16 [*7f*]
						4 [*6h*]	2 [*7g*]
							−3 [*7h*]
Other^a^	−119	−441	−167	75	−257	−45	−80
Total^b^	−4819	−9112	−588	864	−1773	−1456	−1765

a Additional contributions arising from other non-Rh bonding, lone pair, and unassigned occupied orbital contributions, as well as unoccupied orbital contributions. A complete analysis is found in the ESI (Table S9).

b Rounded from sum of contributions at full numerical precision.

c Numbers in parentheses indicate combined contributions from severalequivalent NLMOs.

d The relevant orbitals shown in this work are linear combinations of two NLMOs and were chosen for easier visualization.

**Fig. 5:**
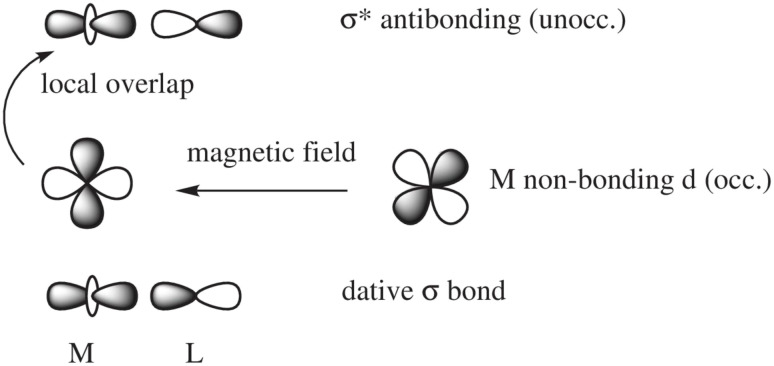
Magnetic-field induced coupling between a nonbonding occupied metal (M) d-orbital and a M–L (L = ligand) antibonding unoccupied σ* orbital.

In a nutshell, the local perturbation of a non-bonding d AO by a magnetic field perpendicular to the page (Fig. 5) effectively creates a function that corresponds to a 45° rotation of the d AO (along with a scaling).^129^ If there is a dative σ-bond (a “donation bond”), then there is a corresponding empty antibonding σ* orbital. If the local symmetry of the rotated non-bonding d AO matches that of the bonding d AO involved in the σ*-bond, as in the figure, the orbitals can couple magnetically. In the theoretical formalism for nuclear magnetic shielding, overlap of magnetic-field perturbed occupied orbitals with unoccupied orbitals of matching local symmetry creates paramagnetic deshielding contributions that are very sensitive to the local AO coefficients and the energy differences between occupied and unoccupied orbitals. These quantities, in turn, depend on the strengths and local symmetries of the donation bonds, and other factors such as the energies and radial extensions of the valence metal d orbitals. Occupied bonding orbitals also contribute to the shielding tensor and serve to modulate the broad shielding trends determined by the number of ligands, the coordination symmetry, and the d-shell occupation.

The two complexes with a full (pseudo-octahedral or *D*_3_-symmetric), six-fold coordination shells have the strongest deshielding from the Rh valence shell. This is consistent with previous research on Pt complexes, where the main difference in the metal shielding in pseudo square-planar *versus* pseudo-octahedral complexes is associated with the paramagnetic induced ring currents in the partially filled metal valence d-shell.^40,128,129^

**Fig. 6:**
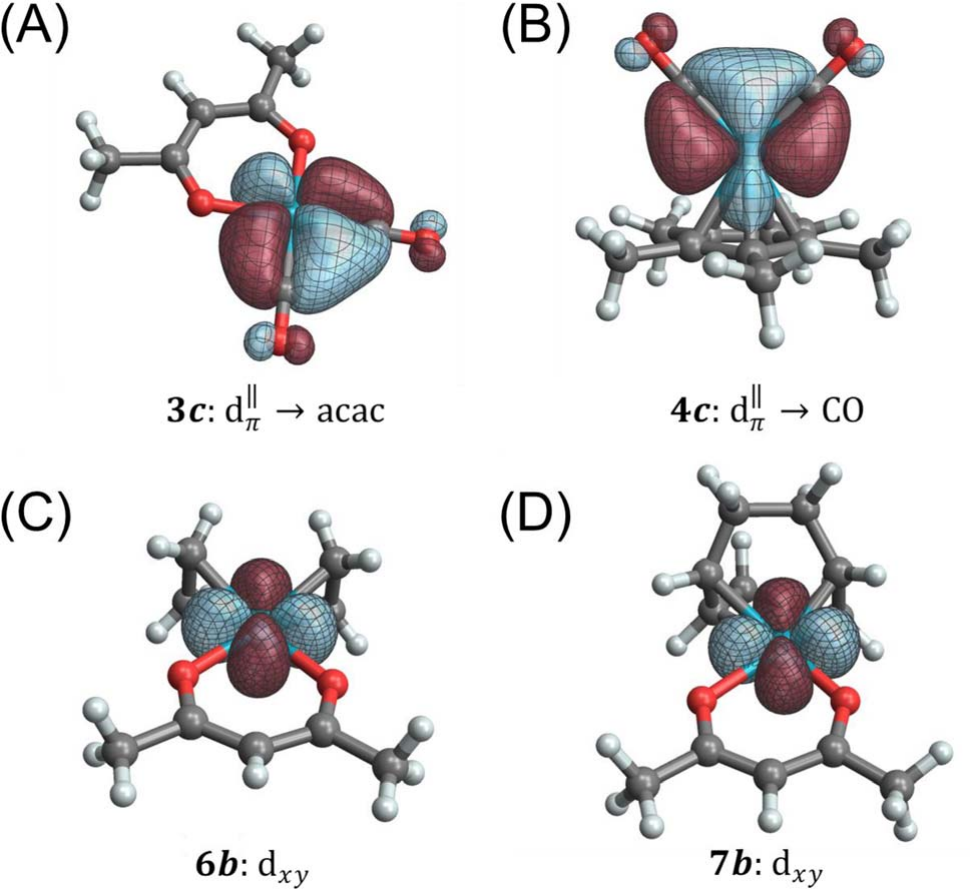
Isosurfaces for 
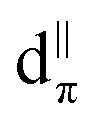
 metal to ligand backbonding orbitals in (A) Rh(CO)_2_(acac) and (B) Rh(CO)_2_Cp*, as well as d_*xy*_ orbitals in (C) Rh(et)_2_(acac), and (D) Rh(cod)(acac) (the *z* axis is perpendicular to the complex plane).

Among the pseudo-square planar complexes, the two carbonyl complexes provide a direct and interesting comparison. The total calculated isotropic shielding for Rh(CO)_2_(acac) is −588 ppm, and for Rh(CO)_2_Cp* it is +864 ppm (a net difference of 1452 ppm). Over 700 ppm of this difference can be attributed to differences in the set of Rh → CO π-backbonding 4*d* orbitals, namely orbitals *3c*/*3d* and *4c*/*4d*, respectively (Fig. 6). In the case of Rh(CO)_2_(acac), applying an in-plane rotation to this orbital results in more direct overlap with unoccupied ligand antibonding orbitals because of the pronounced directionality of the dative σ-bonds and the σ* anti-bonds (in particular, the Rh–O σ* orbitals have the proper symmetry to maximize such overlap); hence, a much larger deshielding effect is observed in comparison to Rh(CO)_2_Cp*. As much as *ca.* 530 ppm of the total shielding difference can be attributed to the contributions from donating NLMOs centered on the acac ligand (*3f*) *versus* those centered on the Cp* ligand (*4e*, *4j*, *4k*), which contribute less and more to shielding, respectively. The shielding effect seen from these latter π orbitals of the aromatic Cp* system is likely associated with an induced ring current. According to this model, this local field reduces the field at the Rh nucleus and therefore leads to an increased shielding.

Table 3 summarizes an analogous NLMO analysis of the calculated shielding span, *Ω*, for the isolated Rh complexes. The span is primarily determined by contributions from the Rh 4d shell, consistent with previous analyses for Pt complexes,^129^ with secondary contributions from overlapping ligand orbitals. The complexes with the broadest spans are those with ligand environments that most depart from Platonic/spherical symmetry, *i.e.*, pseudo-square-planar systems. One departure from this trend is evident from comparison of Rh(CO)_2_(acac) to Rh(et)_2_(acac) and Rh(cod)(acac). The latter complexes, which feature η^2^-π bonding ligands, have a much larger contribution from the in-plane *6b*/*7b* NLMOs (Fig. 6). The corresponding NLMO in Rh(CO)_2_(acac) is *3c*, which was discussed above. In Rh(CO)_2_(acac), backbonding from this orbital results in reduced occupation of the Rh-centered part of the orbital (*i.e.*, reduced occupation of the NLMO's parent NBO). The occupation of the 
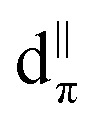
 (*3c*) parent NBO in Rh(CO)_2_(acac) is 1.76 while the parent NBOs of *6b* in Rh(et)_2_(acac) is 1.97 and *7b* in Rh(cod)(acac) is 1.96.

**Table 3:** Summary of NLMO contributions to ^103^Rh shielding span (*Ω* = *σ*_33_ − *σ*_11_) for isolated rhodium complexes (absent crystal embedding)

	[Rh(NH_3_)_5_Cl]^2+^ (1)	Rh(acac)_3_ (2)	Rh(CO)_2_(acac) (3)	Rh(CO)_2_Cp* (4)	[Rh(nbd)Cl]_2_ (5)	Rh(et)_2_(acac) (6)	Rh(cod)(acac) (7)
Rh 4d	350 [*1a*: d_*yz*_]	3352 [*2a*]	−868 [*3a*]	−393 [*4a*]	−686 [*5a*]	−823 [*6a*: d_*z*^2^_]	−1055 [*7a*: d_*z*^2^_]
	−8586 [*1b*: d_*xz*_]	3394 [*2b*]	−62 [*3b*]	−42 [*4b*]	8567 [*5b*]	8827 [*6b*: d_*xy*_]	8269 [*7b*: d_*xy*_]
	8544 [*1c*: d_*xy*_]	−6111 [*2c*]	3627 [*3c* + *3d*]^d^	2618 [*4c*]	249 [*5c*]	227 [*6c*]	
				−840 [*4d*]		−645 [*6d*]	
∑ above 4d^b^	309	635	2697	1343	8130	7585	7213
Rh core	100	−32	406	90	150	163	190
Rh–X	−11 [*1d*(5)]^c^	−74 [*2d*(6)]^c^	289 [*3e*(2)]^c^	305 [*4e*(2)]^c^	−307 [*5d*(2) + *5e*]^c^	−407 [*6e*(2)]^c^	−281 [*7c*(2)]^c^
	−55 [*1e*]	4 [*2e*(3)]^c^	−213 [*3f*(2)]^c^	−85 [*4f*(2)]^c^	−262 [*5f*(4)]^c^	146 [*6f*]	144 [*7d*(2) + *7e*(2)]^c^
						37 [*6g*]	35 [*7f*]
						3 [*6h*]	5 [*7g*]
							6 [*7h*]
Other^a^	−23	27	146	362	470	149	167
Total^b^	319	559	3326	2015	8181	7676	7480

a Additional contributions arising from other non-Rh bonding, lone pair, and unassigned occupied orbital contributions, as well as unoccupied orbital contributions. A complete analysis is found in the ESI (Table S10).

b Rounded from sum of contributions at full numerical precision.

c Numbers in parentheses indicate combined contributions from several equivalent NLMOs.

d The relevant orbitals shown in this work are linear combinations of two NLMOs and were chosen for easier visualization.

**Fig. 7:**
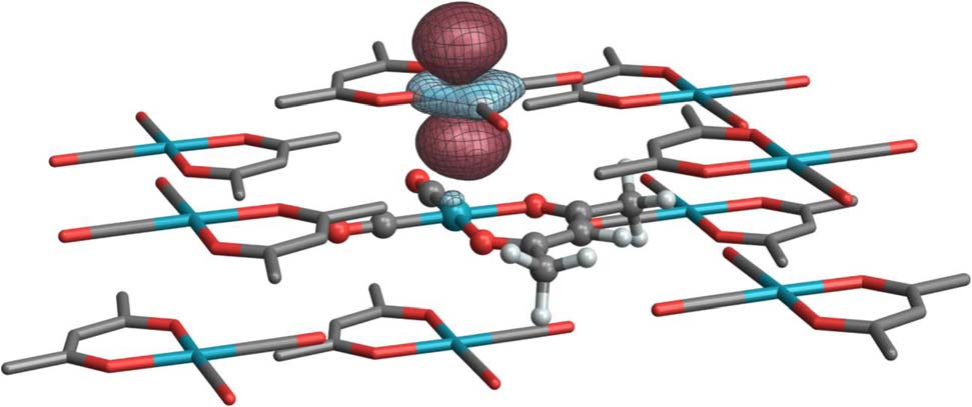
Isosurface of a ‘stacked’ d_*z*^2^_-like NLMO on a neighboring complex in the Rh(CO)_2_(acac) cluster model.

Finally, Table 4 highlights two systems for which crystal embedding most affects the isotropic shielding. The NLMOs listed in Table 4 are assigned labels analogous to those of the corresponding complexes in Table 2 with an added ‘prime’ symbol to indicate they are a part of the same complex in a crystal embedding. In the cluster model, ^103^Rh in both [Rh(NH_3_)_5_Cl]Cl_2_ and Rh(CO)_2_(acac) are further deshielded by 662 ppm and 516 ppm, respectively (relative to the isolated molecule, *cf.*Tables 2 and 4). The former is primarily due to changes in contributions from the *1′a* and *1′b* subshells (the lone Rh–Cl bond defines the *z*-axis). Because of a somewhat different partitioning of low-occupation bonding MOs *versus* antibonding NLMOs in the isolated and embedded systems, an explicit rationale in terms of orbital occupations and magnetic field-induced “rotations” is difficult to obtain from the analysis. However, the set of equatorial Rh–N antibonding orbitals has much higher occupation (0.9) in the clustered system, which would result in weaker paramagnetic coupling in *1′c*, which is consistent with its relatively weaker deshielding effect compared to the other non-bonding d-shell NLMOs. In the case of Rh(CO)_2_(acac), a weak interaction occurs between stacked d_*z*^2^_-like Rh orbitals (Fig. 7), effectively acting as a weak axial ligand, resulting in some additional paramagnetic deshielding from the Rh d-shell. This effect is reminiscent of a previous analysis of stacked Pt complexes.^40^

**Table 4:** Summary of NLMO contributions to ^103^Rh isotropic shielding for rhodium complexes in a cluster-model crystal embedding

	[Rh(NH_3_)_5_Cl]Cl_2_ (1*′*)	Rh(CO)_2_(acac) (3*′*)
Rh 4d	−3149 [*1′a*: d_*yz*_]	−1183 [*3′a*]
	−3182 [*1′b*: d_*xz*_]	−844 [*3′b*]
	−2915 [*1′c*: d_*xy*_]	−1560 [*3′c*]
		−650 [*3′d*]
∑ 4d	−9246	−4237
Rh core	4194	4224
Rh–X	−223 [*1′d*–*h*]	−462 [*3′e*(2)]^c^
	−25 [*1′i*]	−270 [*3′f*(2)]^c^
Other^a^	−182	−359
Total^b^	−5481	−1104

a Additional contributions arising from other non-Rh bonding, lone pair, and unassigned occupied orbital contributions, as well as unoccupied orbital contributions. A complete analysis is found in the ESI (Table S11).

b Rounded from sum of contributions at full numerical precision.

c Numbers in parentheses indicate combined contributions from several equivalent NLMOs.

## 4. Conclusions

The properties of PGE-containing coordination compounds depend critically on metal–ligand interactions; therefore, non-PGE replacements would have to mirror similar interactions, potentially involving unique ligands to compensate for bonding contributions particular to specific PGE–ligand bonds. To date, there are no general, reliable methods for probing the nature of these bonds, which makes searching for replacements a difficult and *ad hoc* process. Herein, we have demonstrated that the combination of ^103^Rh SSNMR spectroscopy and relativistic DFT calculations is a powerful means of characterizing Rh coordination compounds, with the ^103^Rh chemical shift tensor being a robust probe of the nature of Rh–ligand bonding.

The BRAIN-CP pulse sequence, which affords opportunities for broadband excitation, refocusing, and polarization transfer, while also allowing for *T*_2_-based signal enhancement, proves to be invaluable for the acquisition of high signal-to-noise ^103^Rh SSNMR powder patterns. There are also options for indirect detection of ultra-wideline NMR patterns under conditions of MAS using 2D-correlation experiments,^29,138–141^ or under static conditions using the progressive saturation of the ^1^H reservoir (PROSPR) method,^142^ as well at preliminary studies of the use of WURST pulses for ultra-wideline powder patterns under MAS conditions^143,144^ (more of these are in progress in our research group). Together, these methods have potential to aid in the characterization of a plethora of Rh coordination compounds, and will likely find applications in the study of other PGEs such as ruthenium (^99^Ru) and palladium (^105^Pd).

Finally, electronic structure calculations are essential for interpreting the relationships between ^103^Rh chemical shift tensors, electronic structure, and Rh–ligand bonding. To achieve the best agreement with experiment, we find that calculations must (i) include relativistic effects at the spin–orbit level; (ii) be performed using a hybrid exchange–correlation functional when using DFT; and (iii) account for intermolecular interactions that impact ^103^Rh chemical shifts. NLMO and NBO analyses are capable of interpreting chemical shift tensors in terms of contributions from relevant orbitals, providing an additional level understanding of Rh–ligand interactions in terms of the metal- and ligand-centered bonds, lone pairs, and core shells. For the case of these Rh coordination compounds, the large differences in total shielding are primarily determined by the paramagnetic contributions from the Rh 4d shell, as well as ligand orbitals that directly overlap with the Rh valence shells. Such analyses will be very useful for interpreting structure–function–property relationships in PGE-containing compounds, and may even lead to a rational strategy for designing new, advanced materials with tunable physicochemical properties.

## Data availability

All pulse sequences described herein are available online at https://github.com/rschurko. Additional data are available in the ESI.†

## Author contributions

The manuscript was written through contributions from all authors. All authors have given approval to the final version of the manuscript. S. T. H.: conceptualization, formal analysis, investigation, methodology, computation, writing, review, editing. J. S.: investigation, methodology, computation, writing. A. B. P.: conceptualization, formal analysis, investigation, methodology, computation, writing, review, editing. J. J. K.: investigation, methodology, review, editing. S. T.: investigation, methodology, review, editing. A. R. A.: investigation, methodology. Y. X.: investigation, methodology. C. A. O.: investigation, methodology. J. A.: conceptualization, resources, funding, formal analysis, investigation, methodology, computation, writing, review, editing. R. W. S.: conceptualization, resources, funding, formal analysis, investigation, methodology, computation, writing, review, editing.

## Conflicts of interest

There are no conflicts to declare.

## Supplementary Material

SC-015-D3SC06026H-s001

SC-015-D3SC06026H-s002

SC-015-D3SC06026H-s003

SC-015-D3SC06026H-s004

SC-015-D3SC06026H-s005

SC-015-D3SC06026H-s006

SC-015-D3SC06026H-s007

SC-015-D3SC06026H-s008
