# Supplementary material for: Structure and bonding in rhodium coordination compounds: a 103Rh solid-state NMR and relativistic DFT study
Source: Chem Sci. 2023 Dec 7;15(6):2181–96. doi: 10.1039/d3sc06026h (PMC10848688; doi:10.1039/d3sc06026h)
Supplement: SC-015-D3SC06026H-s007 [file SC-015-D3SC06026H-s007.pdf]

Electronic Supplementary Material (ESI) for Chemical Science.
This journal is © The Royal Society of Chemistry 2023

Supporting Information for:

Structure and Bonding in Rhodium Coordination Compounds: A ^{103}Rh Solid-State NMR and Relativistic DFT Study

Sean T. Holmes,[1,2] Jasmin Schönzart,[1,2] Adam B. Philips,[3] James J. Kimball,[1,2] Sara Termos,[1,2] Adam R. Altenhof,[1,2] Yijue Xu,[2] Christopher A. O'Keefe,[4] Jochen Autschbach,[3,*] Robert W. Schurko[1,2*]

[1] Department of Chemistry & Biochemistry, Florida State University, Tallahassee, FL 32306
[2] National High Magnetic Field Laboratory, Tallahassee, FL 32310
[3] Department of Chemistry, University at Buffalo, State University of New York, Buffalo, NY 14260-3000
[4] Department of Chemistry & Biochemistry, University of Windsor, Windsor, ON, Canada N9B 3P4

* Authors to whom correspondence should be addressed
E-mail: rschurko@fsu.edu, jochena@buffalo.edu

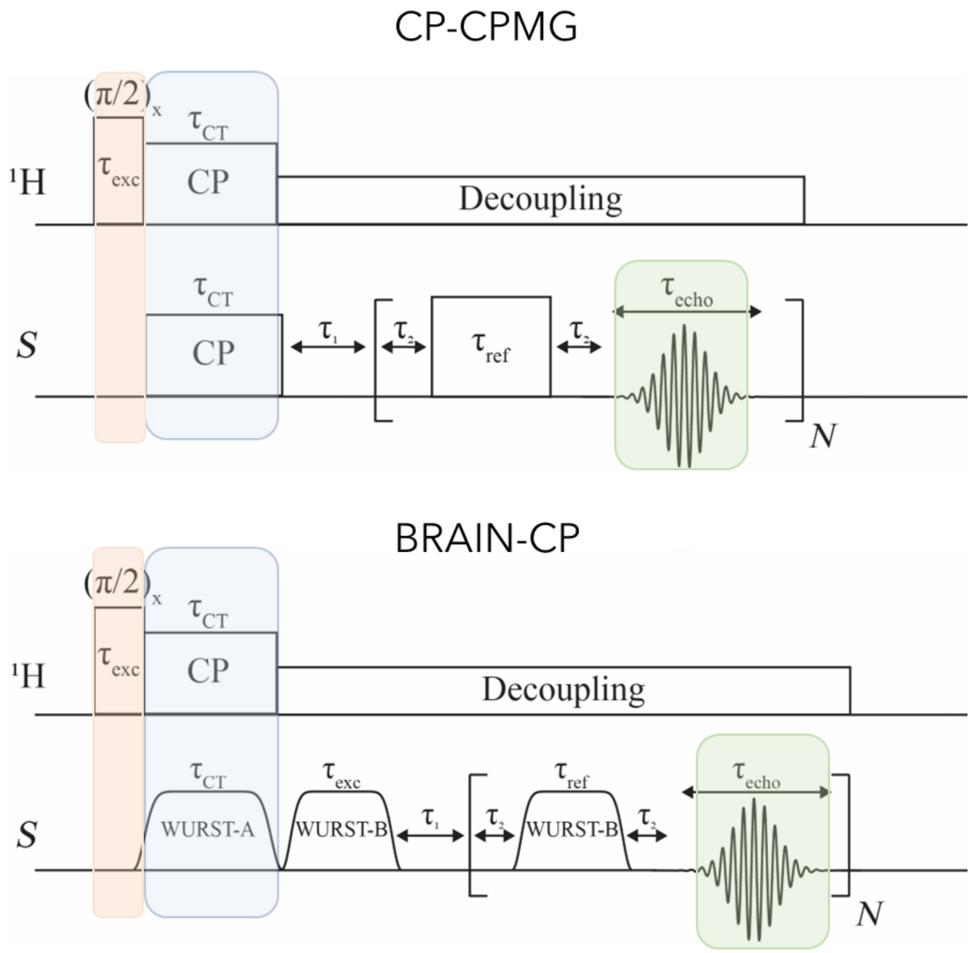

Scheme S1. Illustrations of the CP-CPMG and BRAIN-CP pulse sequences.

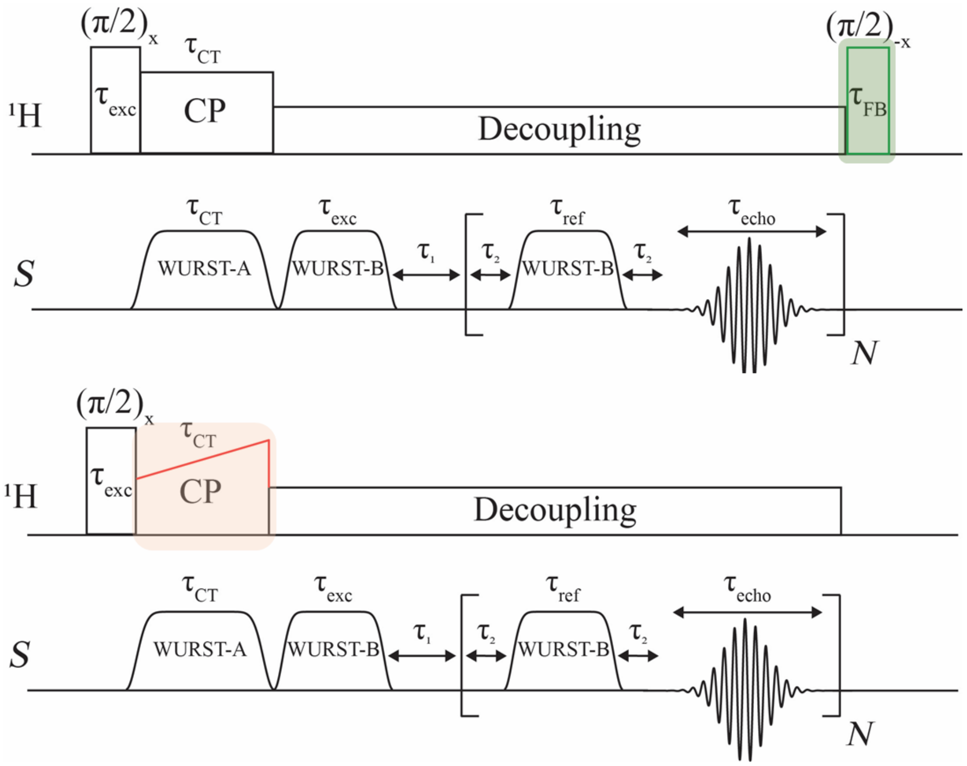

Scheme S2. Illustration of the BRAIN-CP pulse sequence, with modifications highlighted: (i) the inclusion of a flip-back pulse (green box) can potentially reduce the recycle delay; (ii) a ramped-amplitude ^{1}H spin-lock pulse (orange box) compensates for rf inhomogeneities at offsets far from the transmitter.

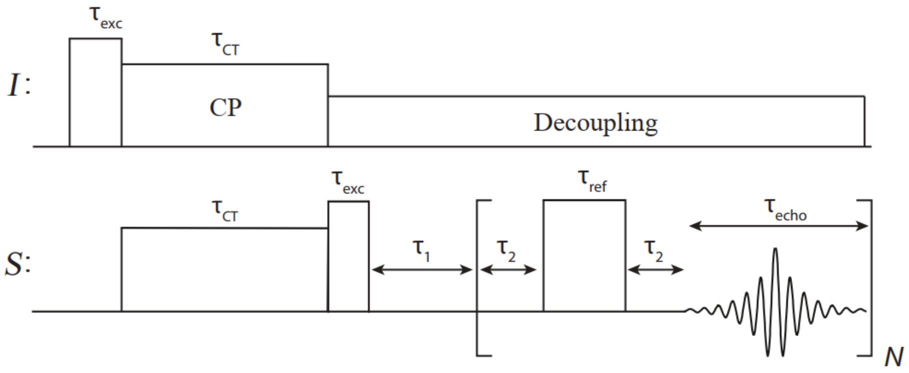

Scheme S3. Illustration of the modified CP-CPMG pulse sequence used for rf calibration.

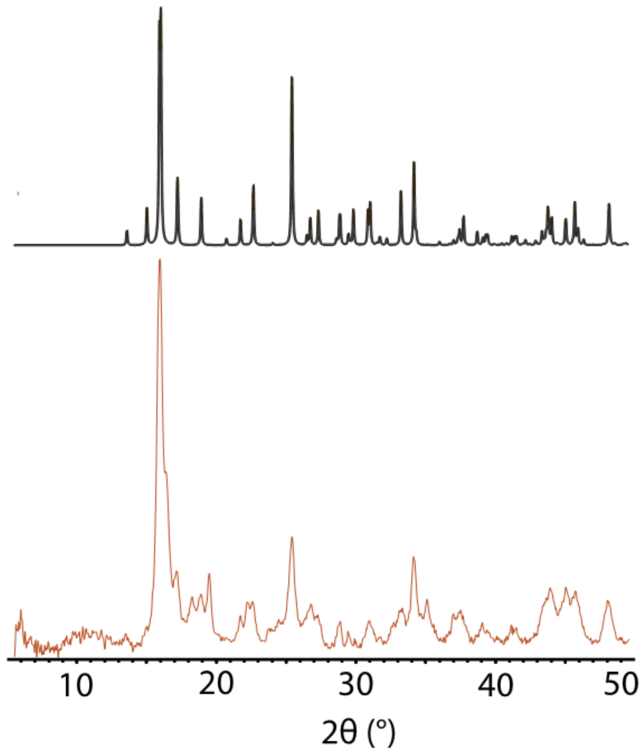

Figure S1. Experimental PXRD pattern of $[Rh(NH_3)_5Cl]Cl_2$ in orange, and corresponding simulation based on the known crystal structure (both at 298 K) in black.

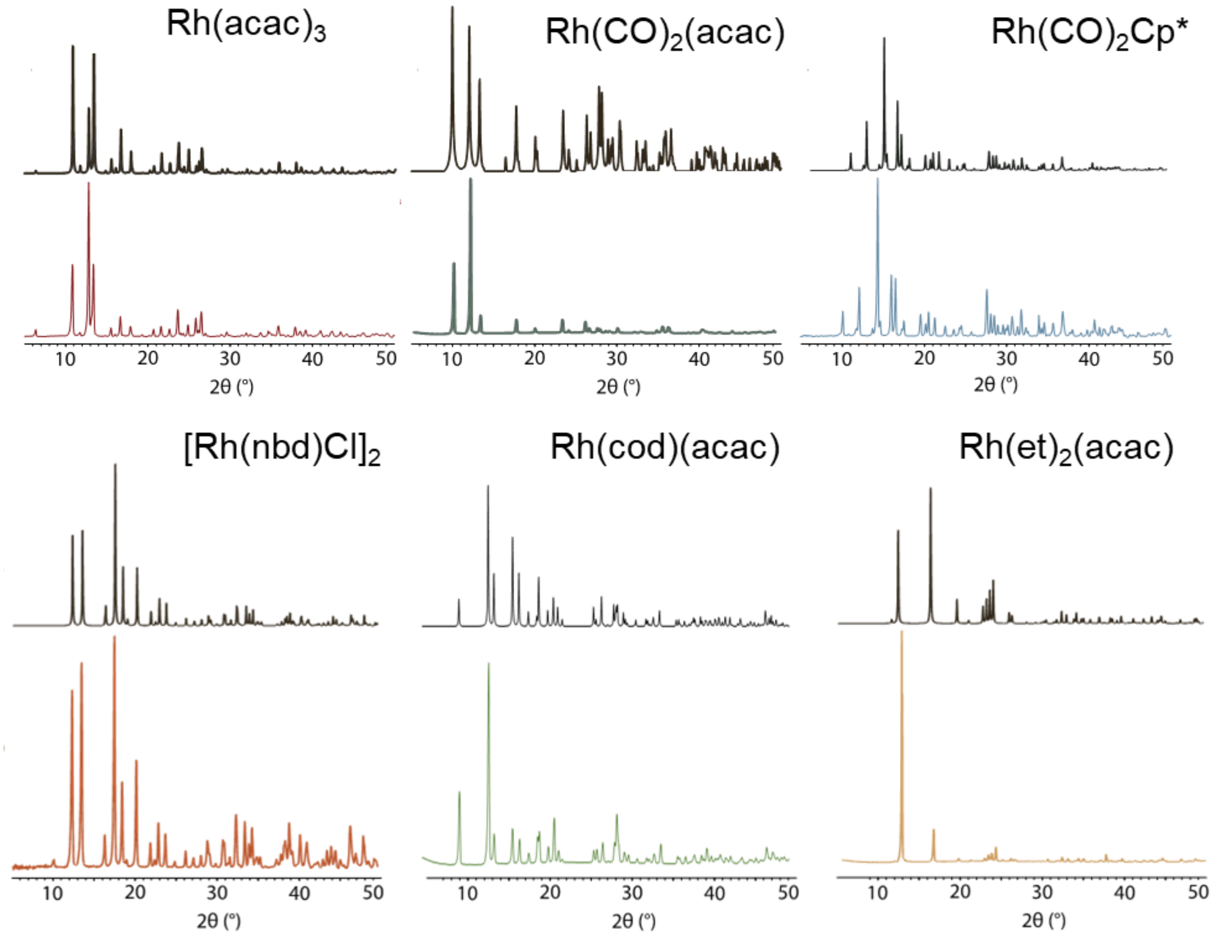

Figure S2. Experimental PXRD patterns (colored lines), and corresponding simulations based on the known crystal structures (black lines). An impurity is marked by as asterisk.

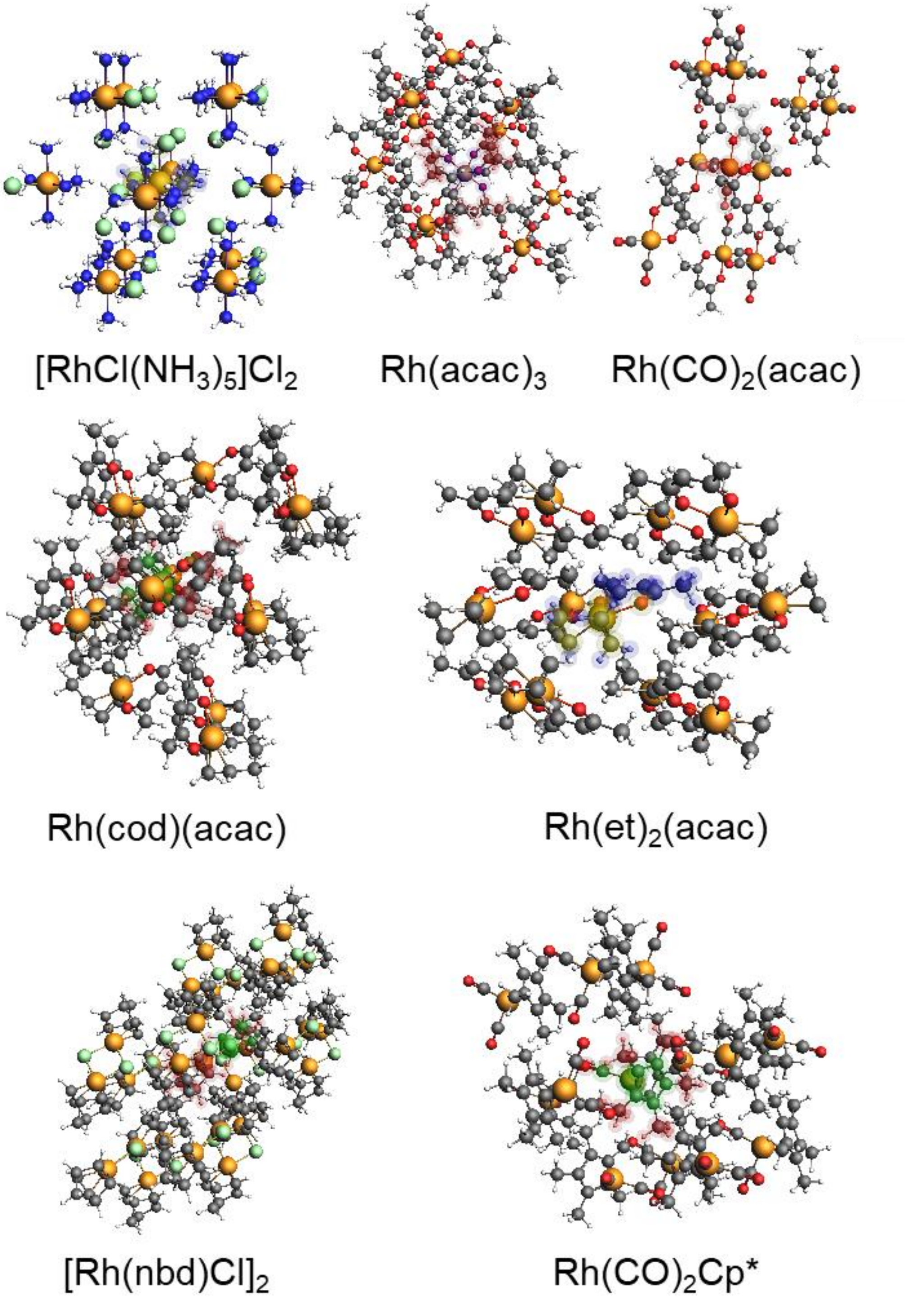

Figure S3. Clusters models used to model the lattice effects on computed ^{103}Rh magnetic shielding tensors.

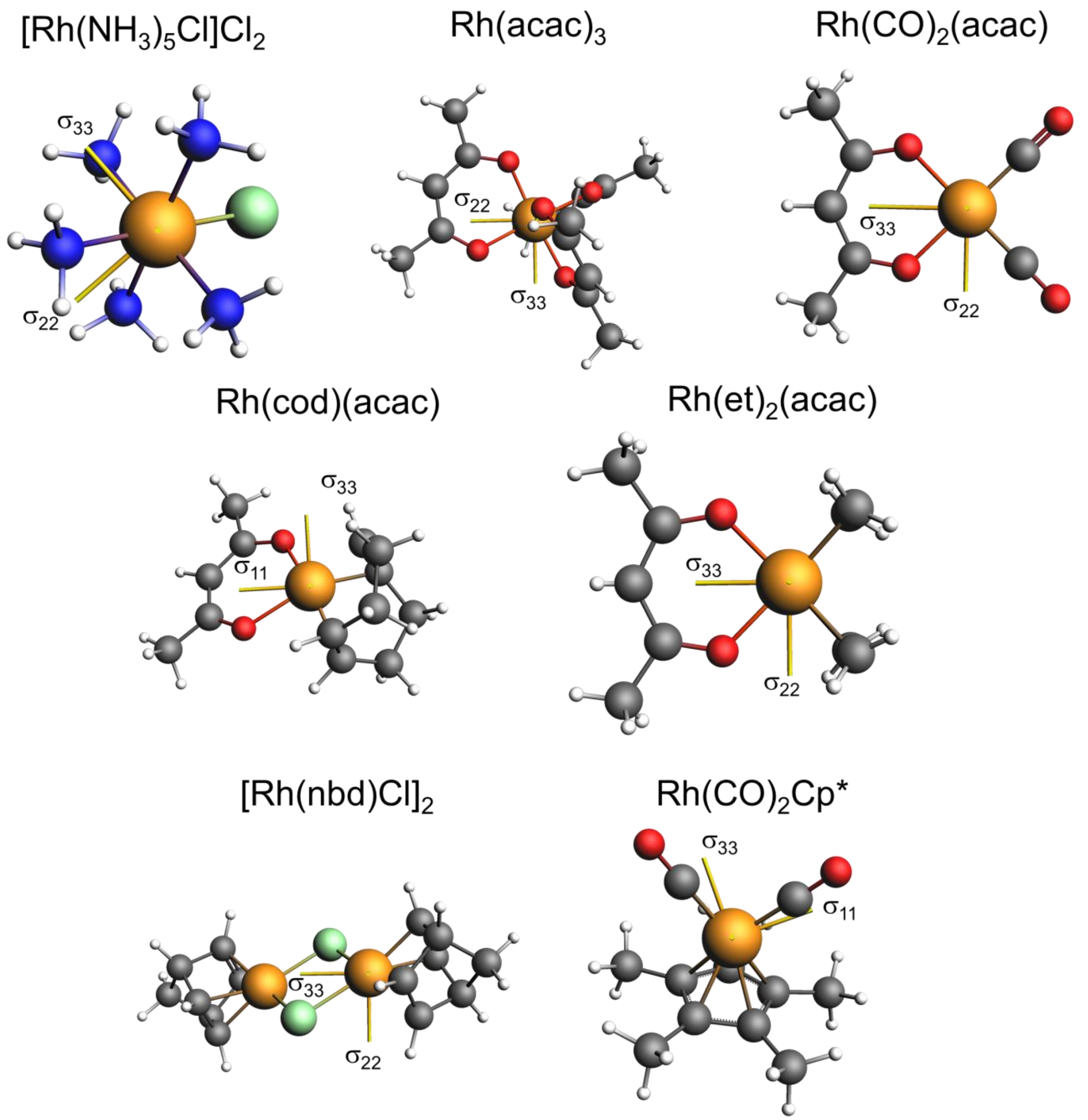

Figure S4. Orientations of the principal values of the ^{103}Rh magnetic shielding tensors, as calculated at the PBE0/SO level. The yellow vectors show the orientations of two principal values, with the third oriented perpendicular to the page.

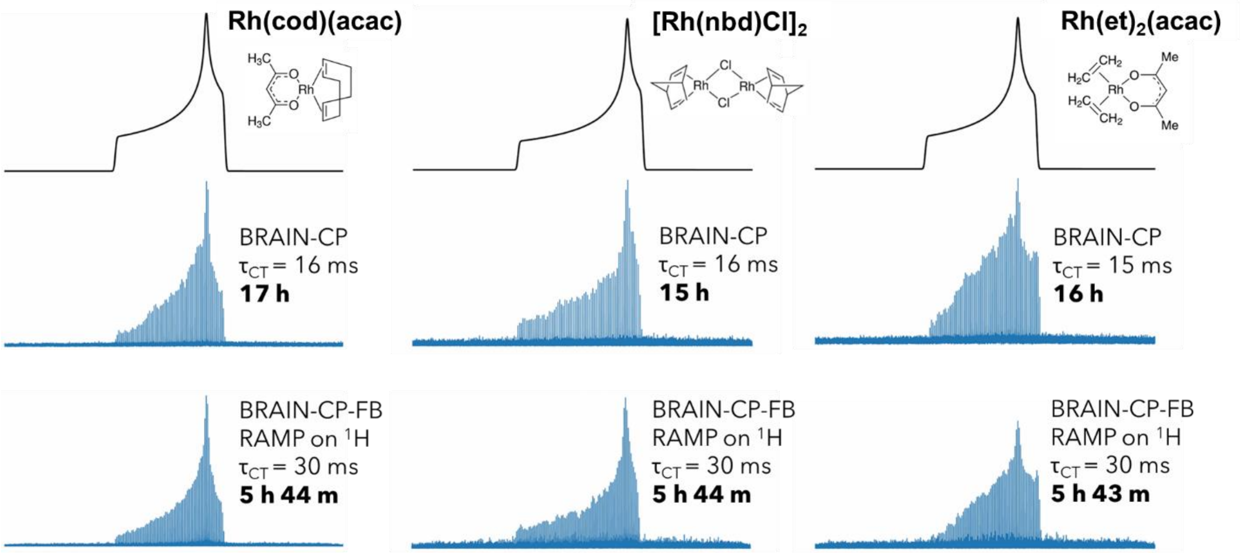

Figure S5. The combination of long contact pulses of 30 ms, ramped-amplitude ^{1}H spin-lock pulses, and flip-back pulses greatly reduces experimental times, relative to experiments acquired using shorter contact pulses (15 – 16 ms), constant-amplitude ^{1}H spin-lock pulses, and no flip-back pulse. All data were acquired at 21.1 T using the BRAIN-CP sequence.

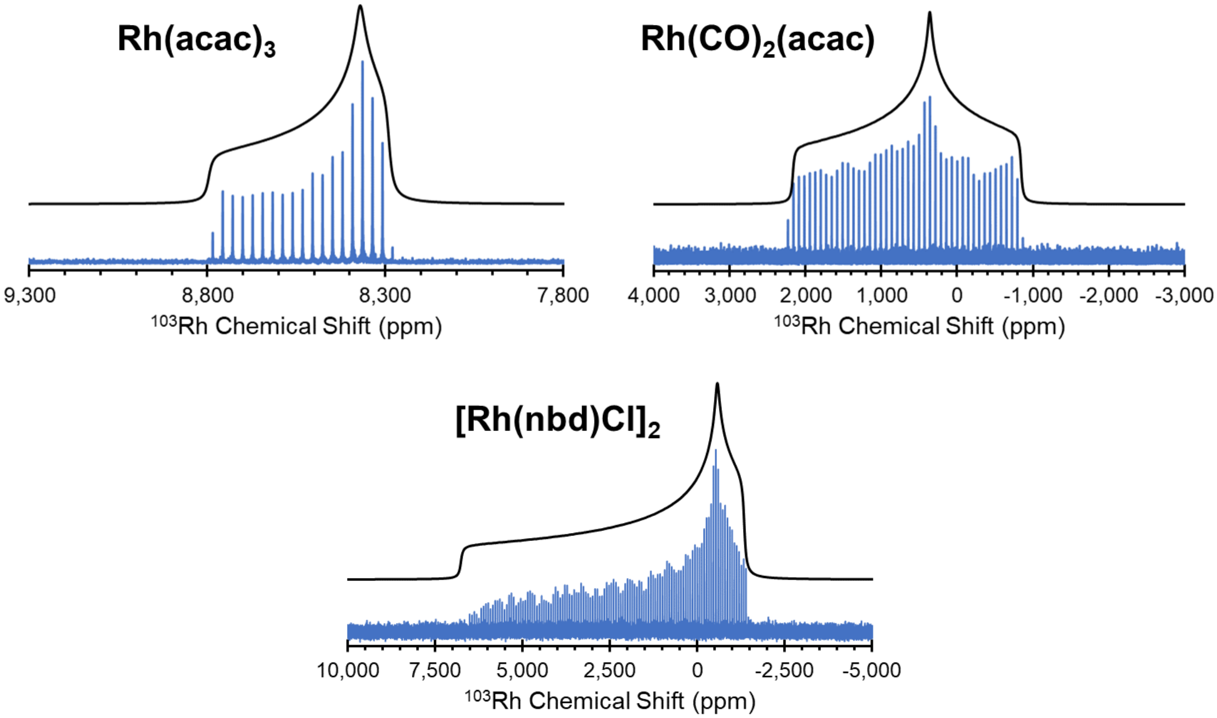

Figure S6. ^{1}H-^{103}Rh BRAIN-CP spectra acquired at 35.2 T for $Rh(acac)_3$, $Rh(CO)_2(acac)$, and $[Rh(nbd)Cl]_2$.

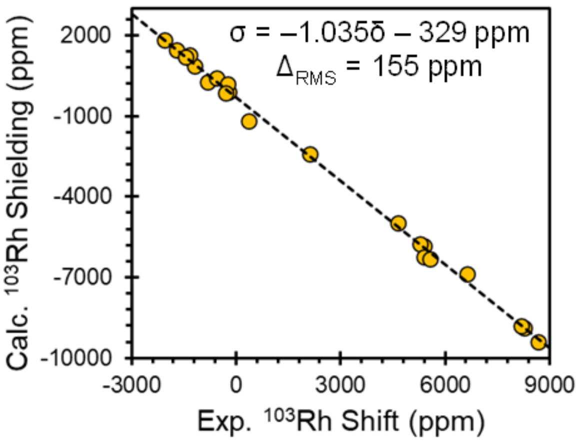

Figure S7. Calculations of ^{103}Rh magnetic shielding tensors using complete first coordination shell clusters of molecules (yellow) or isolated molecules (purple) as structural models. All calculations were performed at the PBE0/SO level. Black lines represent the best fits, with the equations provided.

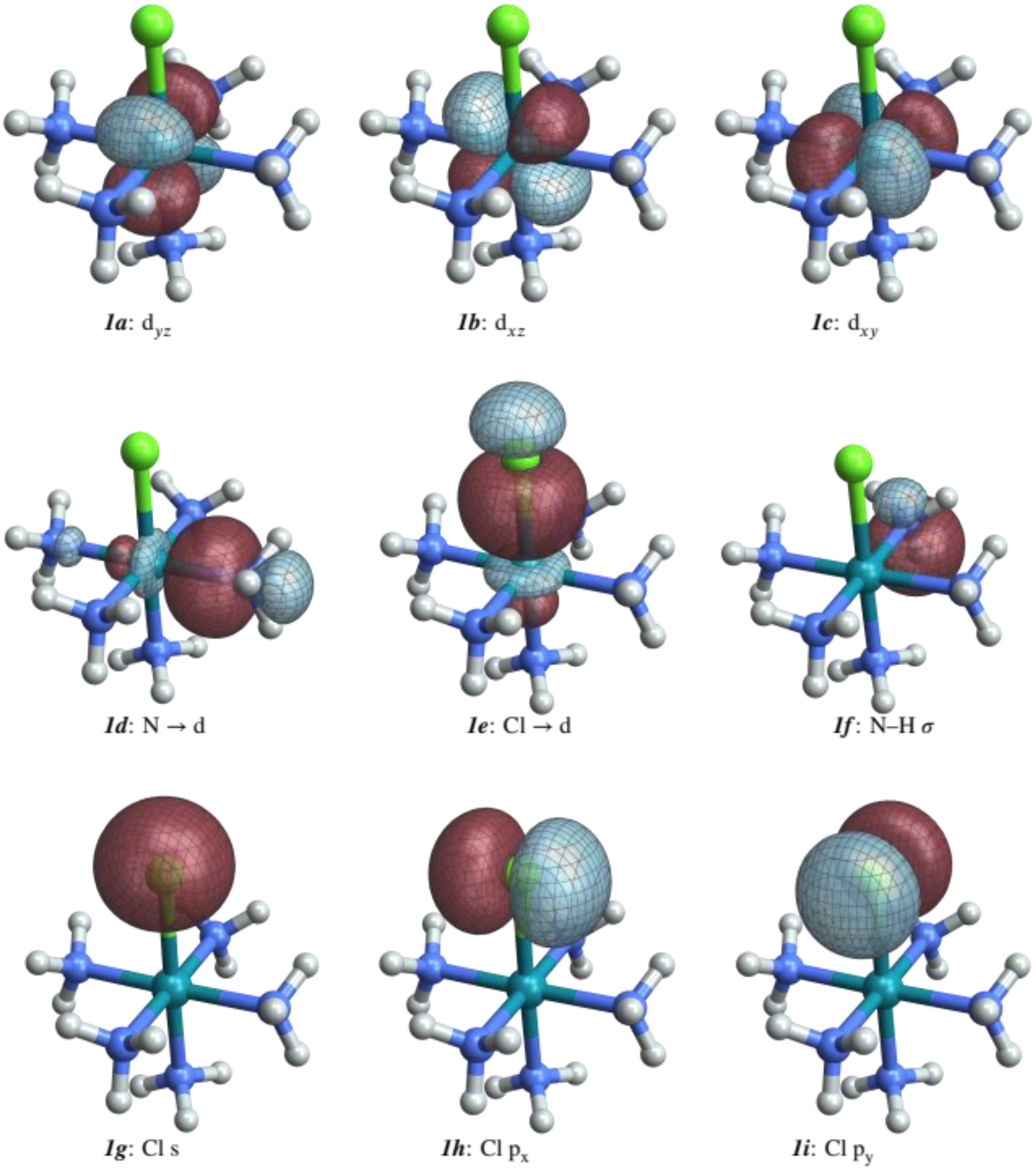

Figure S8. Isosurfaces for primary NLMO contributions to Rh isotropic shielding in $[Rh(NH_3)_5Cl]^{2+}$.

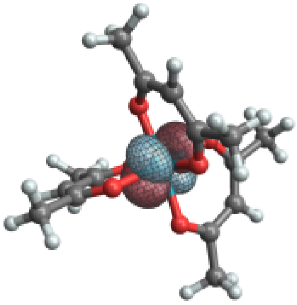

2a: d_{nb}

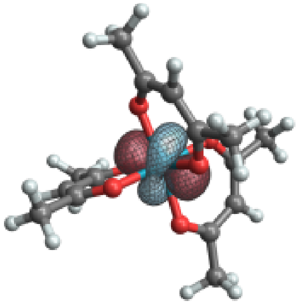

2b: d_{nb}

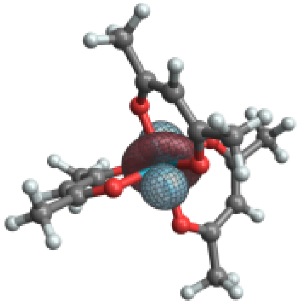

2c: d_{nb}

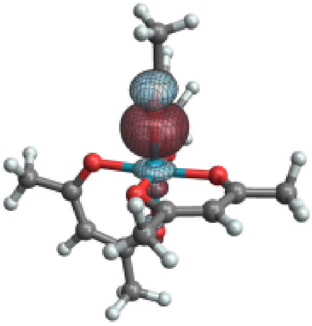

2d: acac O → d

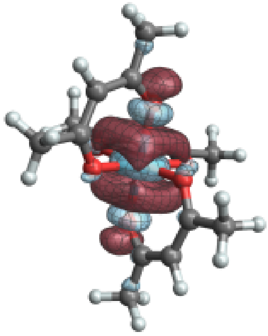

2e: 5s-like σ^*

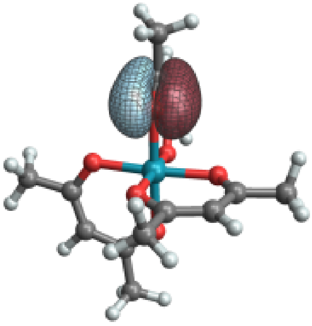

2f: acac C–O π

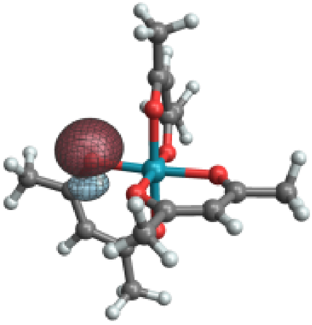

2g: acac O LP

Figure S9. Isosurfaces for primary NLMOs contributing to the Rh isotropic shielding in $Rh(acac)_3$.

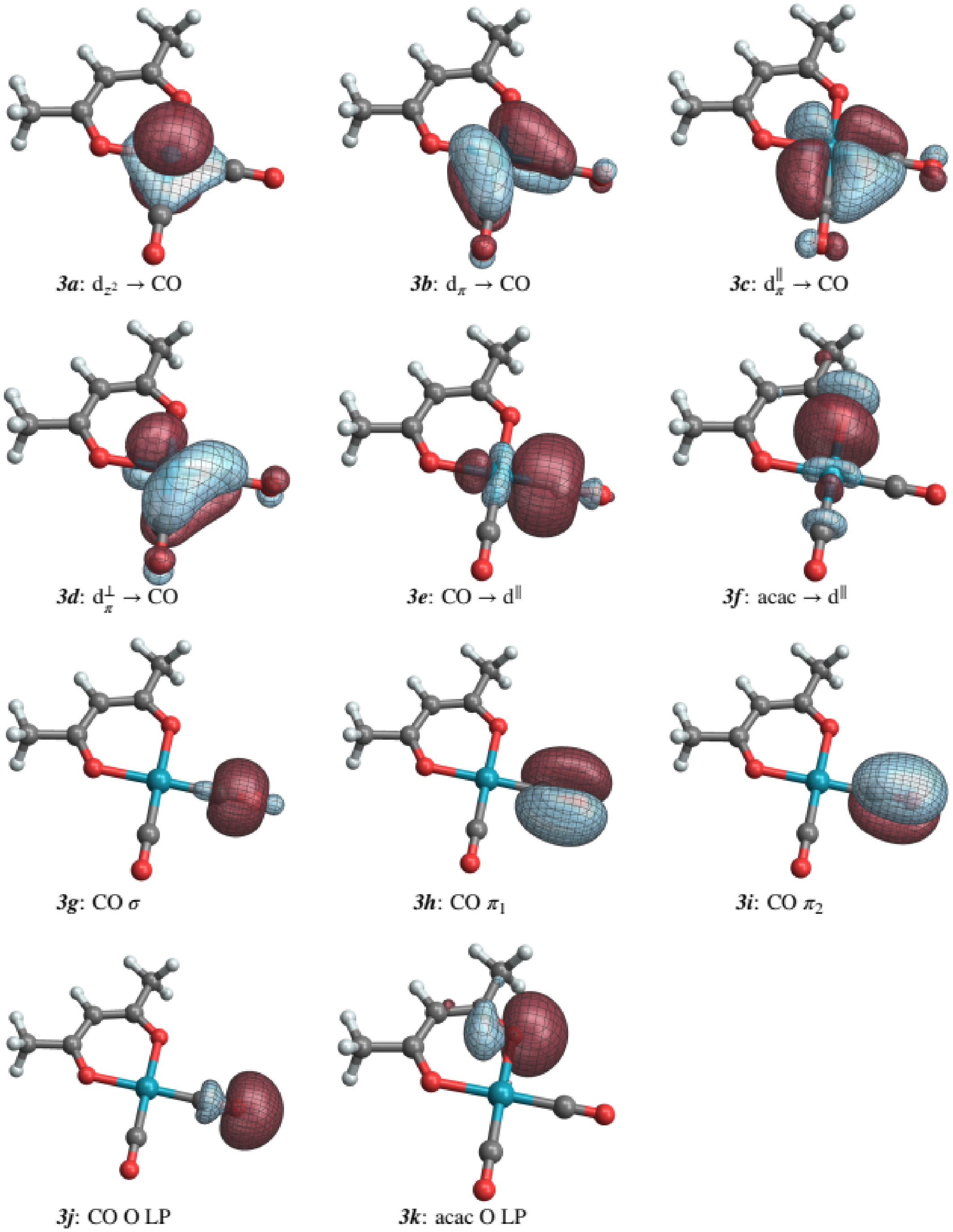

Figure S10. Isosurfaces for primary NLMOs contributing to the Rh isotropic shielding in $Rh(CO)_2(acac)$.

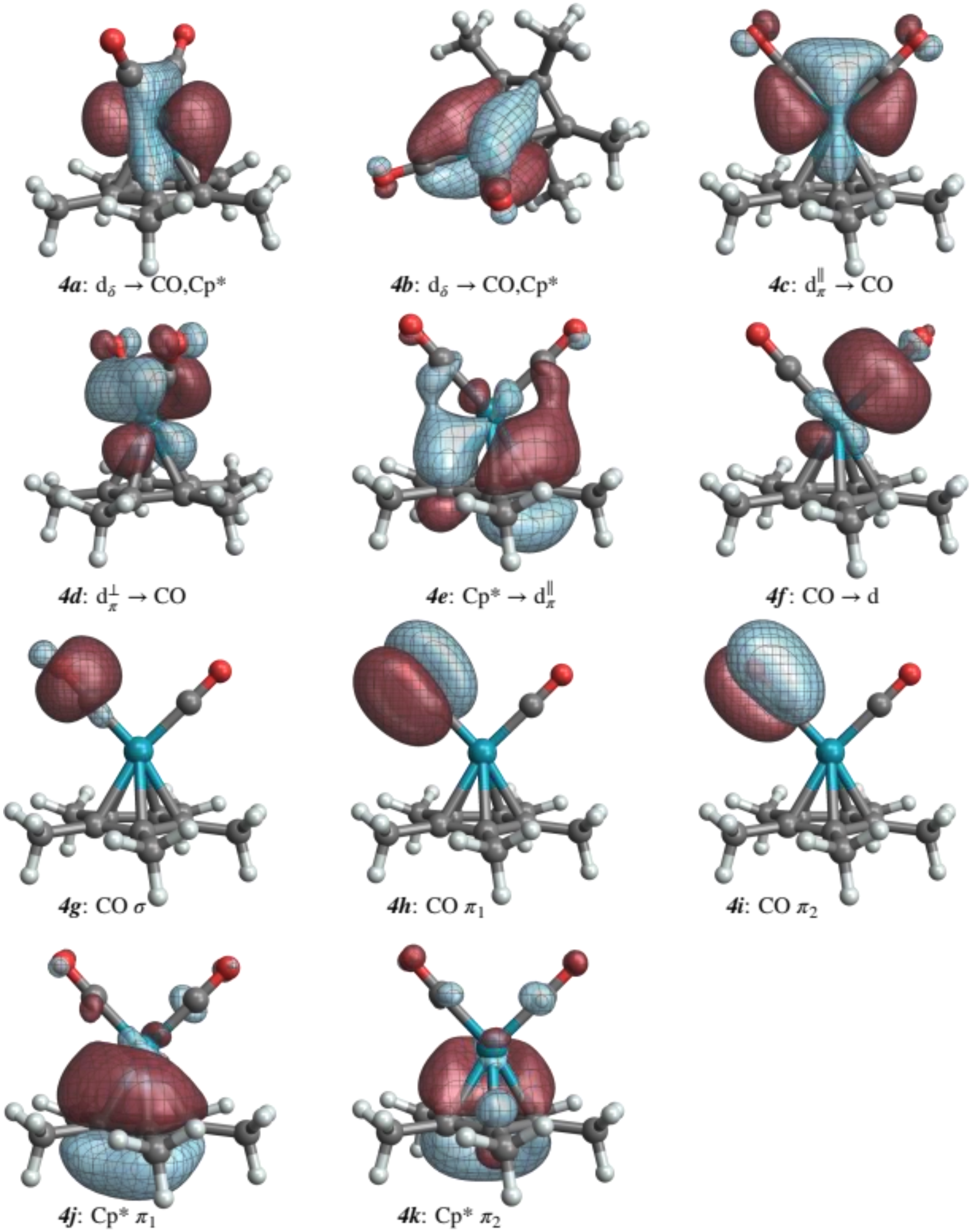

Figure S11. Isosurfaces for primary NLMOs contributing to the Rh isotropic shielding in $Rh(CO)_2Cp^*$.

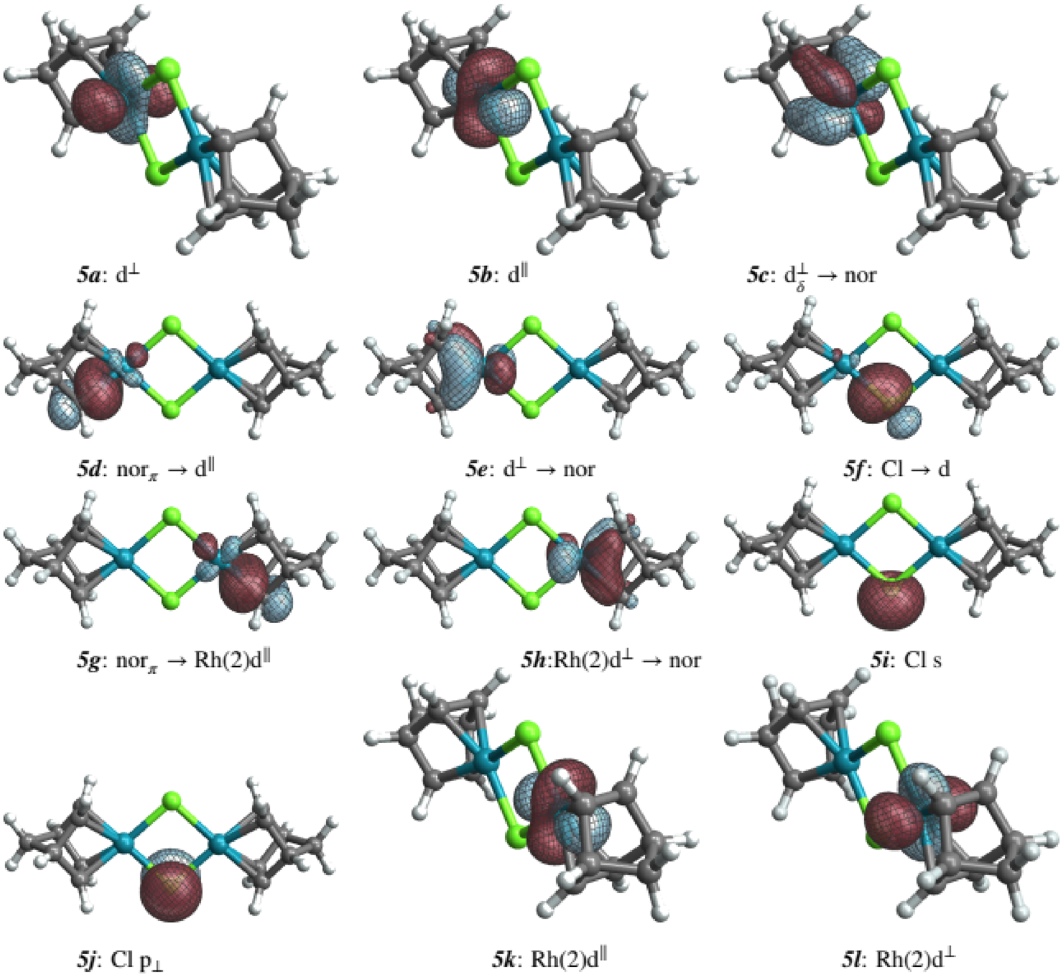

Figure S12. Isosurfaces for primary NLMOs contributing to the Rh isotropic shielding in $[Rh(nbd)Cl]_2$.

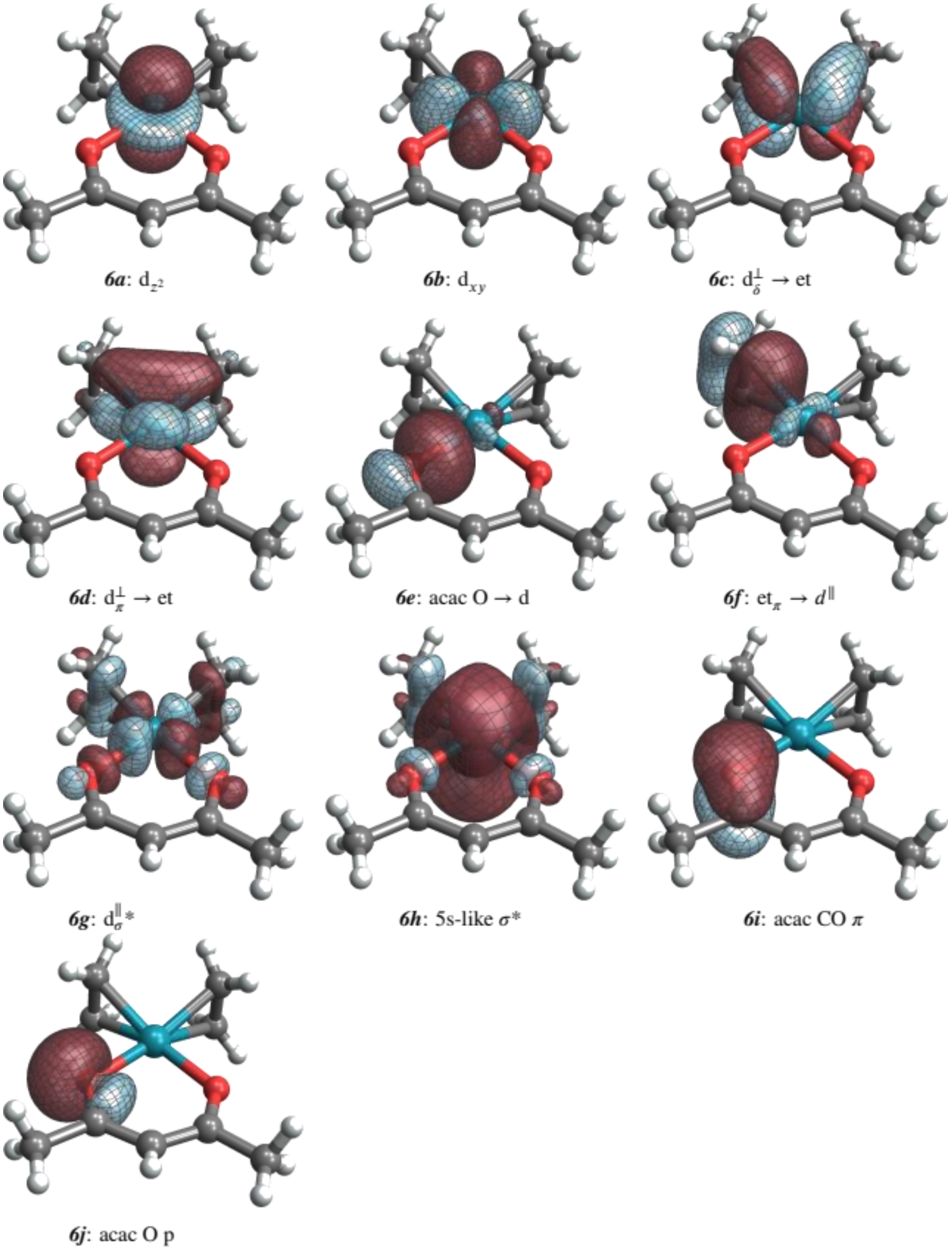

Figure S13. Isosurfaces for primary NLMOs contributing to the Rh isotropic shielding in $Rh(et)_2(acac)$.

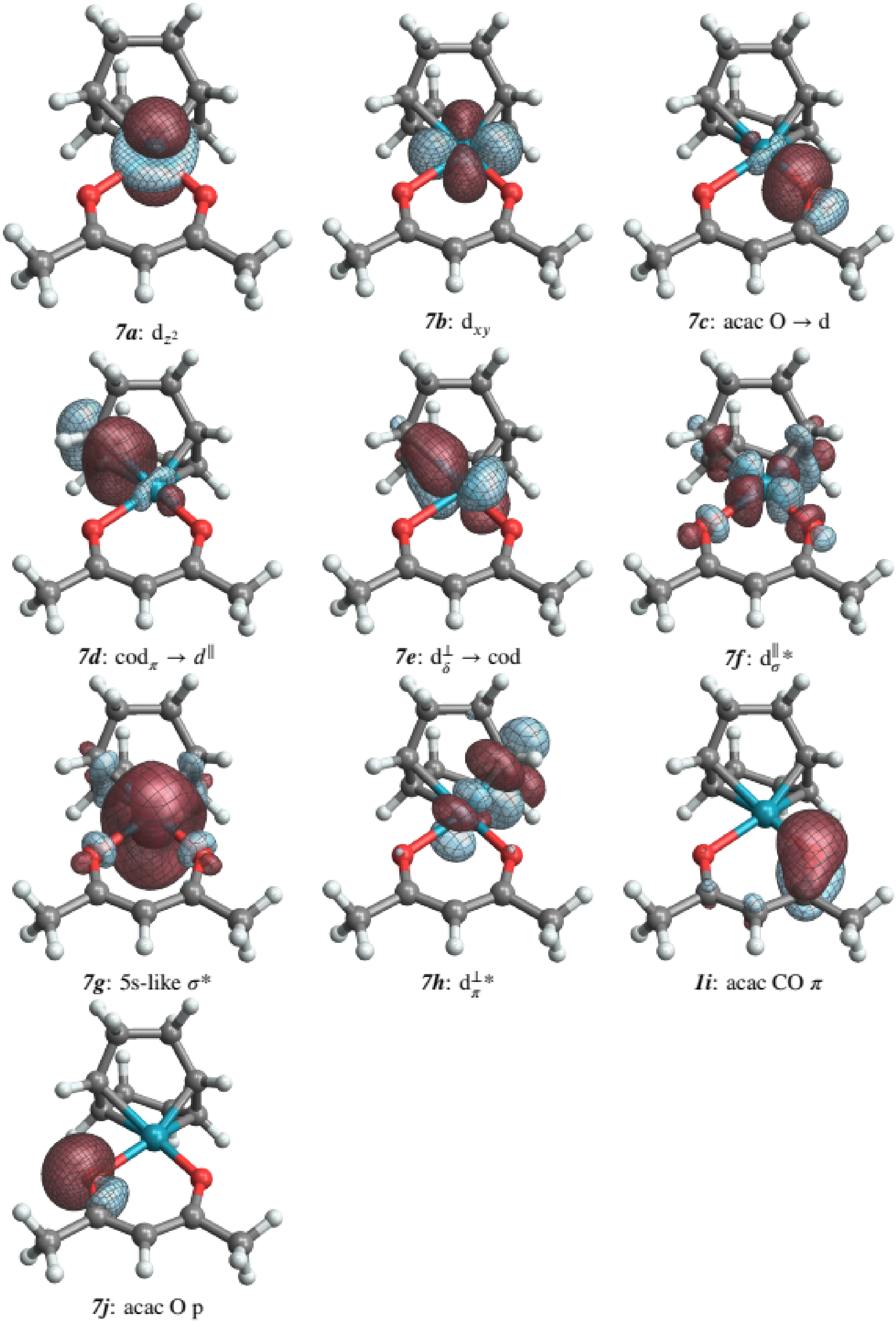

Figure S14. Isosurfaces for primary NLMOs contributing to the Rh isotropic shielding in Rh(cod)(acac).

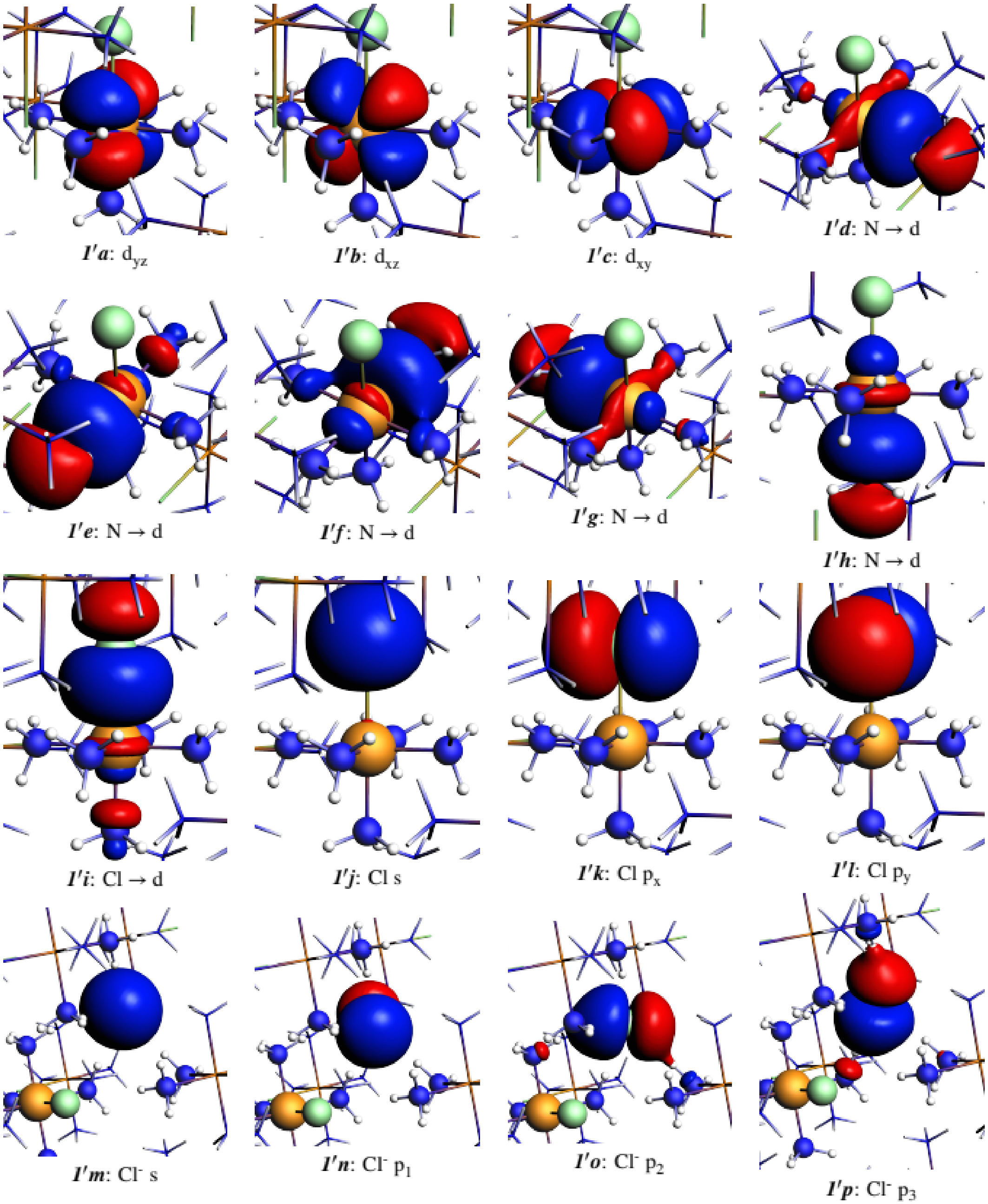

Figure S15. Isosurfaces for primary NLMOs contributing to the Rh isotropic shielding in the cluster model for the $[Rh(NH_3)_5Cl]Cl_2$ complex in the crystal.

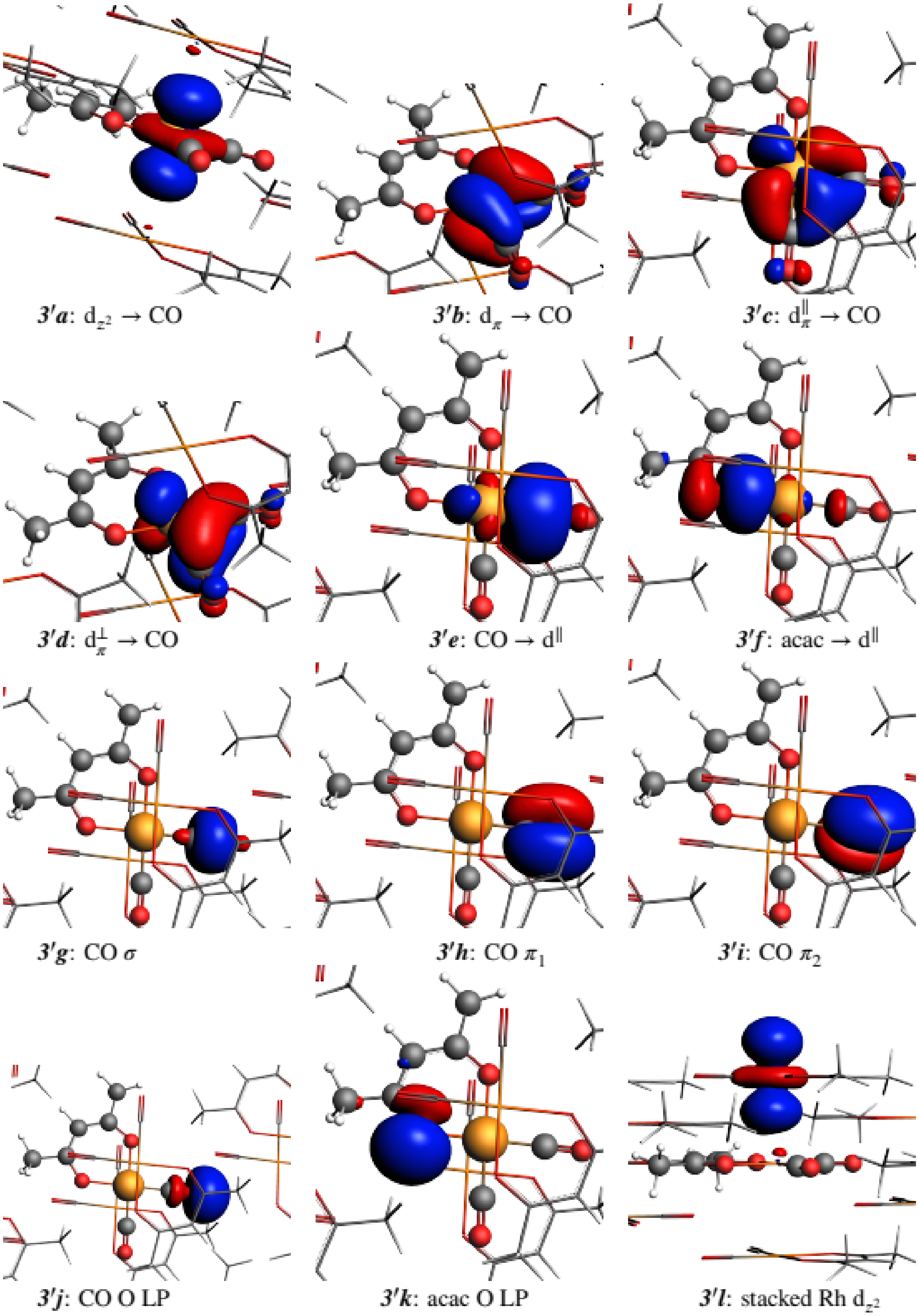

Figure S16. Isosurfaces for primary NLMOs contributing to the Rh isotropic shielding in the cluster model of the $Rh(CO)_2(acac)$ crystal.

Table S1. Some experimental parameters for the ^{1}H-^{103}Rh BRAIN-CP spectra shown in **Figures 1 - 2**.

		$[Rh(NH_3)_5Cl]Cl_2$	$Rh(acac)_3$	$Rh(CO)_2(acac)$	$Rh(CO)_2Cp^*$
Number of Transients	NS	512	1024	1024	4096
Recycle Delay (s)	D1	3	10	30	10
Spectral Window (kHz)	SW	300	100	500	300
Dwell Time (μs)	DW	1.667	5	1	1.667
No. Meiboom-Gill loops	L22	20	19	130	70
Spin Echo Length (μs)	D6	1200	1400	200	300
Acquisition Time (ms)	AQ	34.03	32.5	37.3	27.5
Ring-down delay (μs)	DE	10	10	10	10
^{1}H $\pi/2$ Pulse Length (μs)	P3	4.17	4.17	4.17	4.17
^{1}H $\pi/2$ Pulse rf (kHz)	P13	60	60	60	60
^{1}H CP Pulse rf (kHz)	SP0	25	25	25	25
^{1}H CW Decoupling Field (kHz)	P19	25	25	25	25
WURST-A Length (μs)	P4	10	15	12	16
WURST-A Sweep width (kHz)	Spnam4	50	50	150	150
WURST-A rf (kHz)	SP4	25	25	25	25
WURST-B Length (μs)	P5	25	25	25	25
WURST-B Sweep width (kHz)	Spnam5	50	50	150	150
WURST-B rf (kHz)	SP5	21	14.8	20	20

Table S2. More experimental parameters for the ^{1}H-^{103}Rh BRAIN-CP spectra shown in **Figure 2**.

	$[Rh(nbd)Cl]_2$	$Rh(et)_2(acac)$	Rh(cod)(acac)
Number of Transients	1792	2048	1024
Recycle Delay (s)	30	30	20
Spectral Window (kHz)	625	1000	625
Dwell Time (μs)	0.8	0.5	1.6
No. Meiboom-Gill loops	100	90	50
Spin Echo Length (μs)	200	200	200
Acquisition Time (ms)	31.6	31.8	20.48
Ring-down delay (μs)	10	10	50
^{1}H $\pi/2$ Pulse Length (μs)	4.17	4.17	4.25
^{1}H $\pi/2$ Pulse rf (kHz)	60	60	58.82
^{1}H CP Pulse rf (kHz)	25	25	ramp 22.9 to 18.5
^{1}H CW Decoupling Field (kHz)	25	25	20
WURST-A Length (μs)	16	15	30
WURST-A Sweep width (kHz)	400	400	400
WURST-A rf (kHz)	27	25	20.8
WURST-B Length (μs)	50	50	100
WURST-B Sweep width (kHz)	400	400	400
WURST-B rf (kHz)	24	21	16.4

Table S3. Experimental parameters for the ^{1}H-^{103}Rh BRAIN-CP spectra shown in **Figures 3** and **S4**.

	$Rh(acac)_3$	$Rh(acac)_3$	$Rh(CO)_2(acac)$	$[Rh(nbd)Cl]_2$
Field (T)	21.1	35.2	35.2	35.2
Number of Transients	128	128 or 640 [a]	832	1232
Recycle Delay (s)	10	10	30	30
Spectral Window (kHz)	500	147	500	1500
Dwell Time (μs)	1.00	3.40	1.00	0.333
No. Meiboom-Gill loops	50	40	90	80
Spin Echo Length (μs)	500	700	200	200
Acquisition Time (ms)	35.7	31.6	27.0	25.7
Ring-down delay (μs)	50	50	10.0	10
^{1}H π/2 Pulse Length (μs)	4.17	6.00	6.00	6.00
^{1}H π/2 Pulse rf (kHz)	60	41.67	41.67	41.67
^{1}H CP Pulse rf (kHz)	25	25	25	25
^{1}H CW Decoupling Field (kHz)	25	25	25	25
WURST-A Length (μs)	15	15	12	15
WURST-A Sweep width (kHz)	50	100	300	600
WURST-A rf (kHz)	25	25	25	25
WURST-B Length (μs)	25	25	50	75
WURST-B Sweep width (kHz)	50	100	300	600
WURST-B rf (kHz)	14.8	15.9	20.2	23.4

[a] The spectrum in Figure 3 was acquired with 128 scans, whereas the spectrum in Figure S4 was acquired with 640 scans.

Table S4. Experimental parameters for the ^{1}H-^{103}Rh BRAIN-CP spectra shown in **Figures 4** and **S5**.

	**Rh(cod)(acac)**		**[RhCl(nbd)]$_2$**		**Rh(et)$_2$(acac)**	
	Upper panel	Lower panel	Upper panel	Lower panel	Upper panel	Lower panel
Number of Transients	2048	1024	2048	1792	1024	2048
Recycle Delay (s)	10	20	10	30	20	30
Spectral Window (kHz)	625	1000	625	625	625	1000
Dwell Time (μs)	1.6	0.5	1.6	0.8	1.6	0.5
No. Meiboom-Gill loops	50	90	50	100	50	90
Spin Echo Length (μs)	200	200	200	200	200	200
Acquisition Time (ms)	20.48	31.8	20.48	31.6	20.48	31.8
Ring-down delay (μs)	50	10	50	10	50	10
^{1}H π/2 Pulse Length (μs)	4.25	4.17	4.25	4.17	4.25	4.17
^{1}H π/2 Pulse rf (kHz)	58.82	60	58.82	60	58.82	60
^{1}H CP Pulse rf (kHz)	ramp 23.5 to 18.6	25	ramp 23.2 to 18.4	25	ramp 22.9 to 18.5	25
^{1}H CW Decoupling Field (kHz)	20	25	20	25	20	25
WURST-A Length (μs)	30	15	30	16	30	15
WURST-A Sweep width (kHz)	400	400	400	400	400	400
WURST-A rf (kHz)	20.83	25	20.83	27	20.83	25
WURST-B Length (μs)	100	50	100	50	100	50
WURST-B Sweep width (kHz)	400	400	400	400	400	400
WURST-B rf (kHz)	16.4	21	17.05	24	16.4	21
^{1}H flip-back pulse	yes	no	yes	no	yes	no

Table S5. Crystallographic information and CSD/ICSD codes for all materials.

Material	CSD Code	ICSD Code	Space Group
$[Rh(NH_3)_5Cl]Cl_2$	–	10199	*Pnma*
$Rh(acac)_3$	ACACRH10	–	$P2_1/c$
$Rh(CO)_2(acac)$	ACABRH02	–	$P\bar{1}$
$Rh(CO)_2Cp^*$	COXGUR	–	$P2_1/n$
$[Rh(nbd)Cl]_2$	ZOWVUC	–	$P2_1/c$
Rh(cod)(acac)	COCAR	–	*Cc*
$Rh(et)_2(acac)$	ACDERH01	–	*Pnma*

Table S6. Convergence of GIPAW calculations of ^{103}Rh magnetic shielding tensors with respect to plane-wave cutoff energy and *k*-point spacing in $Rh(CO)_2(acac)$. [a]

Cutoff energy (eV)	*k*-point spacing (Å^{-1})	σ_{11} (ppm)	σ_{22} (ppm)	σ_{33} (ppm)	Energy (eV × 10^{-4})
600	0.08	–1682	–971	122	–1.201085
600	0.07	–1941	–988	83	–1.201088
600	0.06	–1939	–1222	–152	–1.201080
600	0.05	–1939	–1222	–150	–1.201080
500	0.05	–1909	–1229	–170	–1.200814
600	0.05	–1939	–1222	–150	–1.201080
700	0.05	–1945	–1214	–148	–1.201117
800	0.05	–1941	–1212	–146	–1.201121

[a] In these calculations, atomic coordinates were first optimized, followed by calculation of the ^{103}Rh magnetic shielding tensor at the same level.

Table S7. Calculated ^{103}Rh magnetic shielding tensors for isolated molecules using balanced and locally dense basis sets. [a,b]

Material	Basis Set	σ_{11} (ppm)	σ_{22} (ppm)	σ_{33} (ppm)	d_v [c] (ppm)
$[Rh(NH_3)_5Cl]Cl_2$	Balanced	–5069	–4773	–4762	-
	Locally Dense	–5061	–4755	–4745	15
$Rh(acac)_3$	Balanced	–9598	–9068	–9024	-
	Locally Dense	–9478	–8955	–8919	113
$Rh(CO)_2(acac)$	Balanced	–2342	–403	984	-
	Locally Dense	–2374	–388	981	14
$Rh(CO)_2Cp^*$	Balanced	–167	871	1884	-
	Locally Dense	–166	887	1852	14
$[Rh(nbd)Cl]_2$	Balanced	–6936	351	1224	-
	Locally Dense	–6927	381	1239	19
Rh(cod)(acac)	Balanced	–6296	–291	1187	-
	Locally Dense	–6293	–226	1200	32
$Rh(et)_2(acac)$	Balanced	–6154	202	1510	-
	Locally Dense	–6161	265	1524	30

[a] All calculations of ^{103}Rh magnetic shielding tensors were performed at the PBE0/SO level.
[b] Balanced indicates that TZ2P was used for the entire molecule, whereas locally dense indicates that TZ2P was used for the rhodium atom and all directly bonded atoms, and DZ was used for all remaining atoms within the molecule.
[c] The chemical shift distance between the ^{103}Rh magnetic shielding tensors (balanced *vs*. locally-dense basis sets).

Table S8. Summary of calculated ^{103}Rh magnetic shielding tensors.

Material	Method	Structure	σ_{11} (ppm)	σ_{22} (ppm)	σ_{33} (ppm)
$[Rh(NH_3)_5Cl]Cl_2$	GIPAW	Periodic	–4871	–4871	–4114
	PBE/SR	Cluster	–4919	–4900	–4388
	PBE/SO	Cluster	–6201	–3496	–2993
	PBE0/SO	Cluster	–5839	–5755	–4961
	PBE0/SO	Molecule	–5061	–4755	–4745
$Rh(acac)_3$	GIPAW	Periodic	–8114	–7693	–7595
	PBE/SR	Cluster	–8039	–7649	–7500
	PBE/SO	Cluster	–7444	–7044	–6901
	PBE0/SO	Cluster	–9384	–8880	–8810
	PBE0/SO	Molecule	–9478	–8955	–8919
$Rh(CO)_2(acac)$	GIPAW	Periodic	–1948	–1220	–164
	PBE/SR	Cluster	–2131	–1618	–425
	PBE/SO	Cluster	–1705	–1281	–12
	PBE0/SO	Cluster	–2423	–1178	267
	PBE0/SO	Molecule	–2374	–388	981
$Rh(CO)_2Cp^*$	GIPAW	Periodic	–84	685	1554
	PBE/SR	Cluster	–171	556	1393
	PBE/SO	Cluster	249	945	1795
	PBE0/SO	Cluster	–111	865	1829
	PBE0/SO	Molecule	–166	887	1852
$[Rh(nbd)Cl]_2$	GIPAW	Periodic	–8256	972	1875
	PBE/SR	Cluster	–6030	40	580
	PBE/SO	Cluster	–5527	407	999
	PBE0/SO	Cluster	–6866	412	1287
	PBE0/SO	Molecule	–6927	381	1239
Rh(cod)(acac)	GIPAW	Periodic	–5165	–161	450
	PBE/SR	Cluster	–5170	–525	476
	PBE/SO	Cluster	–4705	–113	861
	PBE0/SO	Cluster	–6301	–158	1204
	PBE0/SO	Molecule	–6293	–226	1200
$Rh(et)_2(acac)$	GIPAW	Periodic	–6304	346	1879
	PBE/SR	Cluster	–5160	–233	741
	PBE/SO	Cluster	–4688	140	1147
	PBE0/SO	Cluster	–6248	183	1463
	PBE0/SO	Molecule	–6161	265	1524

Table S9. Summary of NLMO contributions to ^{103}Rh isotropic shielding for isolated rhodium complexes (absent crystal embedding).

	$[Rh(NH_3)_5Cl]Cl_2$<br>(1)	$Rh(acac)_3$<br>(2)	$Rh(CO)_2(acac)$<br>(3)	$Rh(CO)_2Cp^*$<br>(4)	$[Rh(nbd)Cl]_2$<br>(5)	$Rh(et)_2(acac)$<br>(6)	Rh(cod)(acac)<br>(7)
Rh 4d	-2857 [***1a***: d_{yz}]<br>-2846 [***1b***: d_{xz}]<br>-2970 [***1c***: d_{xy}]	-4245 [***2a***]<br>-4253 [***2b***]<br>-3986 [***2c***]	-943 [***3a***]<br>-791 [***3b***]<br>-2134 [***3c***+***3d***][a]	-1007 [***4a***]<br>-581 [***4b***]<br>-947 [***4c***]<br>-463 [***4d***]	-1001 [***5a***]<br>-3451 [***5b***]<br>-523 [***5c***]	-862 [d_{z2}]<br>-3495 [d_{xy}]<br>-580 [***6c***]<br>-366 [***6d***]	-852 [d_{z2}]<br>-3339 [d_{xy}]
∑above 4d	-8673	-12483	-3868	-2998	-4975	-5303	-4191
Rh core	4238	4384	4226	4172	4355	4383	4352
Rh–X	-241 [***1d***(5)][d]<br>-25 [***1e***]	-510 [***2d***(6)][d]<br>-60 [***2e***(3)][d]	-464 [***3e***(2)][d]<br>-316 [***3f***(2)][d]	36 [***4e***(2)] [d]<br>-422 [***4f***(2)][d]	-436 [***5d***(2)+***5e***][a,d]<br>-459 [***5f***(4)][a,d]	-338 [***6e***(2)][d]<br>-140 [***6f***(2)][d]<br>-18 [***6g***]<br>4 [***6h***]	-300 [***7c***(2)][d]<br>-1529 [***7d***(2)+***7e***(2)][a,d]<br>-16 [***7f***]<br>2 [***7g***]<br>-3 [***7h***]
Other Bonding	-30 [***1f***(15)][d]	-140 [***2f***(6)][d]<br>-88 [all acac σ]	-46 [***3g***(2)][d]<br>-25 [***3h***(2)+***3i***(2)][d]	-77 [***4g***(2)+***4h***(2)+***4i***(2)][d]<br>176 [***4j***+***4k***]	-173 [***5g***(2)+***5h***][a,d]	44 [all et σ]<br>-29 [all acac σ]<br>17 [***6i***(2)][d]	4 [all cod σ]<br>-28 [all acac σ]<br>17 [***7i***(2)][d]
Other LP	-8 [Cl s] [***1j***]<br>-5 [Cl p_x+p_y][***1k***+***1l***]	-162 [***2g***(6)][d]	-21 [***3j***(2)][d]<br>-24 [***3k***(2)][d]	-19 [2 CO O]	-20 [***5i***(2)][d]<br>7 [***5j***(2)][d]<br>-12 [***5k***+***5l***]	-33 [***6j***(2)][d]	-35 [***7j***(2)][d]
Other occ.	1	-2	-3	21	12	7	9
Unocc. [b]	-77	-49	-48	-26	-71	-51	-47
Total [c]	-4819	-9112	-588	864	-1773	-1456	-1765

[a] Where indicated, the calculation produced one or more pairs of NLMOs with mixed in-plane vs. out-of-place local π symmetry. For the purpose of visualization, linear combinations of these NLMOs with clean symmetries were generated. The combined shielding contributions from these orbitals are the same for the original NLMOs and the symmetry-adapted linear combinations. In some cases, the contributions are grouped with other NLMOs of matching symmetry.

[b] By construction, the scalar relativistic NLMOs have occupations of either 2 or 0. Contributions shown from unoccupied orbitals come about because of the SO electronic calculation modifying the ground-state density relative to that of the parent scalar relativistic calculation.

[c] Rounded from sum of contributions at full numerical precision.

[d] Numbers in parentheses indicate combined contributions from many equivalent NLMOs.

Table S10. Summary of NLMO contributions to ^{103}Rh shielding span ($\Omega = \sigma_{33} - \sigma_{11}$) for isolated rhodium complexes (absent crystal embedding).

	$[Rh(NH_3)_5Cl]^{2+}$ (**1**)	$Rh(acac)_3$ (**2**)	$Rh(CO)_2(acac)$ (**3**)	$Rh(CO)_2Cp^*$ (**4**)	$[Rh(nbd)Cl]_2$ (**5**)	$Rh(et)_2(acac)$ (**6**)	Rh(cod)(acac) (**7**)
Rh 4d	350 [***1a***: d_{yz}]<br>-8586 [***1b***: d_{xz}]<br>8544 [***1c***: d_{xy}]	3352 [***2a***]<br>3394 [***2b***]<br>-6111 [***2c***]	-868 [***3a***]<br>-62 [***3b***]<br>3627 [***3c***+***3d***]^{a}	-393 [***4a***]<br>-42 [***4b***]<br>2618 [***4c***]<br>-840 [***4d***]	-686 [***5a***]<br>8567 [***5b***]<br>249 [***5c***]	-823 [d_{z2}] [***6a***]<br>8827 [d_{xy}] [***6b***]<br>227 [***6c***]<br>-645 [***6d***]	-1055 [d_{z2}] [***7a***]<br>8269 [d_{xy}] [***7b***]
∑above 4d	309	635	2697	1343	8130	7585	7213
Rh core	100	-32	406	90	150	163	190
Rh–X	-11 [***1d***(5)]^{d}<br>-55 [***1e***]	74 [***2d***(6)]^{d}<br>4 [***2e***(3)]^{d}	289 [***3e***(2)]^{d}<br>-213 [***3f***(2)]^{d}	305 [***4e***(2)]^{d}<br>-85 [***4f***(2)]^{d}	-307 [***5d***(2)+***5e***]^{a,d}<br>-262 [***5f***(4)]^{a,d}	-407 [***6e***(2)]^{d}<br>146 [***6f***]<br>37 [***6g***]<br>3 [***6h***]	-281 [***7c***(2)]^{d}<br>144 [***7d***(2)+***7e***(2)]^{a,d}<br>35 [***7f***]<br>5 [***7g***]<br>6 [***7h***]
Other Bonding	-35 [***1f***(15)]^{d}	82 [***2f***(6)]^{d}<br>9 [all acac σ]	49 [***3g***(2)]^{d}<br>41 [***3h***(2)+***3i***(2)]^{d}	76 [***4g***(2)+***4h***(2)+***4i***(2)]^{d}<br>306 [***4j***+***4k***]	250 [***5g***(2)+***5h***]^{a,d}	122 [all et σ]<br>-15 [all acac σ]<br>21 [***6i***(2)]^{d}	136 [all cod σ]<br>-11 [all acac σ]<br>14 [***7i***(2)]^{d}
Other LP	-12 [Cl s] [***1j***]<br>-45 [Cl p_x+p_y] [***1k***+***1l***]	-67 [***2g***(6)]^{d}	27 [***3j***(2)]^{d}<br>28 [***3k***(2)]^{d}	25 [2 CO O]	-25 [***5i***(2)]^{d}<br>16 [***5j***(2)]^{d}<br>33 [***5k***+***5l***]	26 [***6j***(2)]^{d}	25 [***7j***(2)]^{d}
Other occ.	-1	-3	-29	-70	114	-35	-28
Unocc. ^{b}	1	6	30	25	82	30	32
Total ^{c}	319	559	3326	2015	8181	7676	7480

^{a} Where indicated, the calculation produced one or more pairs of NLMOs with mixed in-plane vs. out-of-place local π symmetry. For the purpose of visualization, linear combinations of these NLMOs with clean symmetries were generated. The combined shielding contributions from these orbitals are the same for the original NLMOs and the symmetry-adapted linear combinations. In some cases, the contributions are grouped with other NLMOs of matching symmetry.
^{b} By construction, the scalar relativistic NLMOs have occupations of either 2 or 0. Contributions shown from unoccupied orbitals come about because of the SO electronic calculation modifying the ground-state density relative to that of the parent scalar relativistic calculation.
^{c} Rounded from sum of contributions at full numerical precision.
^{d} Numbers in parentheses indicate combined contributions from many equivalent NLMOs.

Table S11. Summary of NLMO contributions to ^{103}Rh isotropic shielding for rhodium complexes in a cluster-model crystal embedding.

	$[Rh(NH_3)_5Cl]Cl_2$ (**1′**)	$Rh(CO)_2(acac)$ (**3′**)
Rh 4d	-3149 [***1′a***: d_{yz}]<br>-3182 [***1′b***: d_{xz}]<br>-2915 [***1′c***: d_{xy}]	-1183 [***3′a***]<br>-844 [***3′b***]<br>-1560 [***3′c***]<br>-650 [***3′d***]
∑ 4d	-9246	-4237
Rh core	4194	4224
Rh–X	-223 [***1′d–h***]<br>-25 [***1′i***]	-462 [***3′e***(2)][c]<br>-270 [***3′f***(2)][c]
Other Bonding	-62 [N–H(15)] [c]	-44 [***3′g***(2)][c]<br>-27 [***3′h***(2)+***3′i***(2)][c]
Other LP	-8 [Cl s] [***1′j***]<br>-45 [***1′k***(8)+***1′l***(8)][c]<br>22 [***1′m–p*** (8)][c]	-21 [***3′j***(2)][c]<br>-31 [***3′k***(2)][c]<br>-88 [***3′l***(2)]
Other occ.	-7	-99
Unocc. [a]	-82	-49
Total [b]	-5481	-1104

[a] By construction, the scalar relativistic NLMOs have occupations of either 2 or 0. Contributions shown from unoccupied orbitals come about because of the SO electronic calculation modifying the ground-state density relative to that of the parent scalar relativistic calculation.
[b] Rounded from sum of contributions at full numerical precision.
[c] Numbers in parentheses indicate combined contributions from that many equivalent NLMOs.

Supplement 1: Chemical Shift Distance. The relationship between calculated principal components of the ^{103}Rh magnetic shielding tensors ($\sigma_{ii}^{v,\mathrm{calc}}$) and experimental principal components of ^{103}Rh chemical shift tensors ($\delta_{ii}^{v,\mathrm{exp}}$) is described by the following expression:

$$\sigma_{ii}^{v,\mathrm{exp}} = A\delta_{ii}^{v,\mathrm{calc}} + B. \tag{1}$$

where the index *v* denotes the rhodium site (*v* = 1, 2, …, *N*), the index *i* denotes the principal component of the shielding tensor (*i* = 1, 2, 3), *A* represents the slope of the correlation line, and *B* represents the interpolated shielding of the reference state. Calculated chemical shifts ($\delta_{ii}^{v,\mathrm{calc}}$) are derived from the following expression:

$$\delta_{ii}^{v,\mathrm{calc}} = (B - \sigma_{ii}^{v,\mathrm{calc}})/A. \tag{2}$$

The *chemical shift distance* for atom *v*, d_v, provides a comparison between a calculated and experimental chemical shift tensor with a single scalar value in ppm. Given two sets of principal components of chemical shift tensors, d_v is defined by the following expression:

$$\begin{aligned} d_v = \Big(\frac{1}{15}\Big[&3\big(\delta_{11}^{v,\mathrm{calc}} - \delta_{11}^{v,\mathrm{exp}}\big)^2 + 3\big(\delta_{22}^{v,\mathrm{calc}} - \delta_{22}^{v,\mathrm{exp}}\big)^2 \\ &+ 3\big(\delta_{33}^{v,\mathrm{calc}} - \delta_{33}^{v,\mathrm{exp}}\big)^2 \\ &+ 2\big(\delta_{11}^{v,\mathrm{calc}} - \delta_{11}^{v,\mathrm{exp}}\big)\big(\delta_{22}^{v,\mathrm{calc}} - \delta_{22}^{v,\mathrm{exp}}\big) \\ &+ 2\big(\delta_{11}^{v,\mathrm{calc}} - \delta_{11}^{v,\mathrm{exp}}\big)\big(\delta_{33}^{v,\mathrm{calc}} - \delta_{33}^{v,\mathrm{exp}}\big) \\ &+ 2\big(\delta_{22}^{v,\mathrm{calc}} - \delta_{22}^{v,\mathrm{exp}}\big)\big(\delta_{33}^{v,\mathrm{calc}} - \delta_{33}^{\mathrm{exp}}\big)\Big]\Big)^{1/2}. \end{aligned} \tag{3}$$

A root-mean-square chemical shift distance for an ensemble of *N* chemical shift tensors (Δ_{RMS}) is determined by the following expression:

$$\Delta_{\mathrm{RMS}} = \left(\frac{1}{N}\sum_{v=1}^{N} d_v^2\right)^{1/2}. \tag{4}$$
